# Effects of (music-based) rhythmic auditory cueing training on gait and posture post-stroke: A systematic review & dose-response meta-analysis

**DOI:** 10.1038/s41598-019-38723-3

**Published:** 2019-02-18

**Authors:** Shashank Ghai, Ishan Ghai

**Affiliations:** 1 Institute of Sports Science, Leibniz University Hannover, Hannover, Germany; 2 Rsgbiogen, New Delhi, India

## Abstract

Gait dysfunctions are common post-stroke. Rhythmic auditory cueing has been widely used in gait rehabilitation for movement disorders. However, a consensus regarding its influence on gait and postural recovery post-stroke is still warranted. A systematic review and meta-analysis was performed to analyze the effects of auditory cueing on gait and postural stability post-stroke. Nine academic databases were searched according to PRISMA guidelines. The eligibility criteria for the studies were a) studies were randomized controlled trials or controlled clinical trials published in English, German, Hindi, Punjabi or Korean languages b) studies evaluated the effects of auditory cueing on spatiotemporal gait and/or postural stability parameters post-stroke c) studies scored ≥4 points on the PEDro scale. Out of 1,471 records, 38 studies involving 968 patients were included in this present review. The review and meta-analyses revealed beneficial effects of training with auditory cueing on gait and postural stability. A training dosage of 20–45 minutes session, for 3–5 times a week enhanced gait performance, dynamic postural stability i.e. velocity (Hedge’s g: 0.73), stride length (0.58), cadence (0.75) and timed-up and go test (−0.76). This review strongly recommends the incorporation of rhythmic auditory cueing based training in gait and postural rehabilitation, post-stroke.

## Introduction

Stroke is the second main cause of disability across the world^1,2^. Stroke related disability substantially affects activities of daily living^3^, promotes dependency^4^, social isolation^5^, and a poorer quality of life^6^. Physical manifestations in patients affected from stroke are usually exhibited on the contralateral side of the affected brain region^7^. However, independent to the site of lesion paralytic changes, cognitive dysfunctions, and sensory impairments are also observed in most of the cases^8^. Despite advancements in modern rehabilitation approaches poor prognosis for motor recovery post-stroke is still prevalent^9^, especially for recovering gait^10^, and postural stability^11^. Studies suggest that gait functionality is an important predictor for determining the health status outcome and quality of life in stroke patients^12^.

Best practice principles in stroke rehabilitation indicate that effective stroke interventions should be individually-tailored, meaningful, task-specific, involve sufficient repetition and challenge to induce recovery^13–15^. Training with rhythmic auditory cueing has the potential to meet such guidelines while yielding improvements in motor function^16,17^. Literature suggests that the efficacy and specificity of training with auditory cueing relies on the reinforcement of auditory-motor functional connectivity in related brain systems^16–19^. Consequentially, increased motor cortex excitability in the affected hemisphere and enhancement of motor recovery on the affected side is observed^20–22^. Likewise, neuroimaging studies outlining a time frame for establishing auditory-motor co-activations have suggested that such training can utilize the intricate auditory-motor functional connectivity and instigate motor (re)learning efficiently as compared to conventional approaches^23–26^. A recent dose-response meta-analysis by Ghai^17^ has also substantiated these findings. The author reported considerable enhancements in arm function post-stroke after training with auditory cueing in in sessions lasting between 30 min to 1-hour. Despite this compelling evidence, a joint consensus concerning the influence of auditory cueing-based therapy and effective training dosages for recovering gait post-stroke are still warranted.

To the best of our knowledge, five systematic reviews and meta-analyses till date, have evaluated the effects of rhythmic auditory cueing on gait recovery post-stroke^27–31^. Even though, all of the included reviews reported beneficial effects of auditory cueing on gait performance, we observed substantial methodological limitations in these reviews: a) A limited number of controlled clinical trials were included b) The search for the studies was performed across few academic databases c) Ambiguity in the meta-analysis approach was observed i.e. no sub-group analysis or heterogeneity tests were performed d) The search for relevant literature was limited to few languages. Therefore, interpretation of results from these reviews from both a qualitative and quantitative perspective might indicate a bias. Moreover, till date, no meta-analysis has synthesized the current state of literature for determining specific training dosages with rhythmic auditory cueing for recovering gait and postural stability post-stroke. Therefore, in this present systematic review and meta-analysis an attempt has been made to address these shortfalls, by focusing on two main objectives:

1. Analyze the influence of training with rhythmic auditory cueing on spatiotemporal gait and postural stability parameters in individuals post-stroke.
2. Determine appropriate training dosages with auditory cueing that allows substantial enhancements in gait and postural stability.

Findings from this review shall help augment the predictive power concerning a patient’s response to auditory cueing interventions, thereby guiding researchers, clinicians and patients themselves in their choice of an optimal rehabilitation intervention.

## Methods

This review was conducted according to the guidelines outlined by Preferred Reporting Items for Systematic Reviews and Meta-analysis: The PRISMA statement^32^. A PRISMA checklist has been provided in Supplementary Table 3.

### Data sources and search strategy

Nine academic databases were searched from inception until December 2017: Web of science, PEDro, EBSCO, Scopus, MEDLINE, Indian citation index, Cochrane central register of controlled trials, EMBASE and PROQUEST. A sample PICOS search strategy for EMBASE academic database has been provided (Table 1).

**Table 1:** Sample search strategy EMBASE.

PICOS	Databse	Embase
	Date	10/12/2017
	Strategy	#1 and #2 and #3 and #4 and #5 and #6
**P**	**#1**	(‘Stroke’ OR ‘Apoplexy’ OR ‘CVA’ OR ‘Cerebral Stroke’ OR ‘Cerebrovascular accident’ OR ‘Cerebrovascular Accident, Acute’ OR ‘ABI’ OR ‘Acquired brain injury’ OR ‘Cerebrovascular Apoplexy’ OR ‘Cerebrovascular Stroke’ OR ‘Stroke, Acute’ OR ‘Stroke, sub-acute’ OR ‘Stroke, chronic’ OR ‘Vascular Accident, Brain’ OR ‘Hemiplegia, Crossed’ OR ‘Hemiplegia, Flaccid’ OR ‘Hemiplegia, Spastic’ OR ‘Hemiplegia, Transient’ OR ‘Monoplegia’ OR ‘Lower Extremity Paresis’ OR ‘Muscular Paresis’ OR ‘Muscle Paresis’ OR ‘Monoparesis’ OR ‘Hemiparesis’)/de OR (Stroke OR Apoplexy OR CVA OR Cerebral Stroke OR Cerebrovascular accident OR Cerebrovascular Accident, Acute OR ABI OR Acquired brain injury OR Cerebrovascular Apoplexy OR Cerebrovascular Stroke OR Stroke, Acute OR Stroke, sub-acute OR Stroke, chronic OR Vascular Accident, Brain OR Hemiplegia, Crossed OR Hemiplegia, Flaccid OR Hemiplegia, Spastic OR Hemiplegia, Transient OR Monoplegia OR Lower Extremity Paresis OR Muscular Paresis OR Muscle Paresis OR Monoparesis OR Hemiparesis):ti,ab
**I**	**#2**	(‘rhythmic auditory cueing’ OR ‘rhythmic auditory cueing’ OR ‘rhythmic acoustic cueing’ OR ‘rhythmic auditory entrainment’ OR ‘metronome cueing’ OR ‘metronome’ OR ‘rhythmic metronome cueing’ OR ‘acoustic stimulus’ OR ‘acoustic cueing’ OR ‘acoustic cueing’ OR ‘external stimuli’ OR ‘external cueing’ OR ‘external cueing’ OR ‘music therapy’ OR ‘Neurological music therapy’ OR ‘tempo’ OR ‘beat’ OR ‘rhythm’ OR ‘RAC’ OR ‘NMT’ OR ‘real-time auditory cueing’ OR ‘sonification’)/de OR (rhythmic auditory cueing OR rhythmic auditory cueing OR rhythmic acoustic cueing OR rhythmic auditory entrainment OR metronome cueing OR metronome OR rhythmic metronome cueing OR acoustic stimulus OR acoustic cueing OR acoustic cueing OR external stimuli OR external cueing OR external cueing OR music therapy OR Neurological music therapy OR tempo OR beat OR rhythm OR RAC OR NMT OR real-time auditory cueing OR sonification)ti,ab
**C**	**n/a**	n/a
**O**	**#3**	(‘walking’ OR ‘gait’ OR ‘locomotion’ OR ‘range of motion’ OR ‘ROM’ OR ‘ambulation’ OR ‘mobility’ OR ‘treadmill gait’ OR ‘balance’ OR ‘stability’ OR ‘stride’ OR ‘gait training’ OR ‘gait rehabilitation’ OR ‘postural stability’ OR ‘posture’ OR ‘dynamic posture’ OR ‘dynamic balance’ OR ‘static posture’ OR ‘static balance’ OR ‘balance’)/de OR (walking OR gait OR locomotion OR range of motion OR ROM OR ambulation OR mobility OR treadmill gait OR balance OR stability OR stride OR gait training OR gait rehabilitation OR postural stability OR posture OR dynamic posture OR dynamic balance OR static posture OR static balance OR balance);ti,ab
**S**	**#4**	(‘intervention study’ OR ‘cohort analysis’ OR ‘longitudinal study’ OR ‘cluster analysis’ OR ‘crossover trial’ OR ‘cluster analysis’ OR ‘randomized trial’ OR ‘major clinical study’)/de OR (longitudinal OR cohort OR crossover trial OR cluster analysis OR randomized trial OR clinical trial OR controlled trial);ti,ab
	**#5**	(‘rehabilitation’ OR ‘treatment’ OR ‘rehab’ OR ‘management’ OR ‘therapy’ OR ‘physiotherapy’ OR ‘physical therapy’ OR ‘prevention’ OR ‘risk prevention’)/de OR (rehabilitation OR treatment OR rehab OR management OR therapy OR physiotherapy OR physical therapy OR prevention OR risk prevention);ti,ab
	**#6**	(‘age groups’ OR ‘adolescent’ OR ‘young’ OR ‘elderly’ OR ‘old’ AND (‘gender’ OR ‘male’ OR ‘female’)/de OR (age groups OR adolescent OR young OR elderly OR old AND (gender OR male OR female));ti;ab

An inclusion criterion was determined by two reviewers (S.G, I.G) for the systematic review procedure. The inclusion criterion for the studies were (i) The studies were either randomized controlled trials, cluster randomized controlled trials or controlled clinical trials (ii) The studies evaluated music-based auditory cueing interventions (any training duration, treatment setting) (iii) The studies evaluated spatiotemporal gait parameters (gait velocity, cadence, stride length, stride time, single/double-limb support duration)^33^ (iv) The studies evaluated static or dynamic aspects of postural stability (Berg balance scale, Fugl-Meyer lower body assessment, Timed-up and go test, Timed sit-to-stand test, Activity-specific balance confidence scale)^34^ (v) The studies included a subjective analysis of stroke outcome (optional) (vi) The studies scored ≥4 points on PEDro quality scale (studies scoring <3 considered of “poor” quality with high risk of biasing excluded^35^) (vii) The studies were conducted on human participants affected from stroke (any age, disease duration and type) (viii) The studies were published in peer-reviewed academic journals or conference proceedings (un-published “grey” literature was not included) (ix) The studies were published in English, German, Hindi, Punjabi or Korean languages.

The two reviewers (S.G, I.G) duplicated the study selection, data extraction and quality assessment of the included studies. After selection of the articles, following data were extracted from each study i.e. author, journal name, publication year, selection criteria for participants, total sample size, description of the participants (gender, age, health status, duration of stroke, comorbidities), applied treatment intervention, characteristics of applied auditory stimuli, treatment interventions for the control group, dual-task application (if any), outcome measures, results, conclusions and special notes by authors. The data were then summarized and tabulated (Supplementary Table 2). In case of lack of quantitative data in the manuscript, the reviewers (S.G, I.G) made attempts to contact respective corresponding authors for data.

### Quality and risk of bias assessment

The quality of the reviewed studies was assessed using the PEDro methodological quality scale^36^. This quality scale consists of 11 items which address external validity, internal validity, and interpretability. The scale can effectively detect potential bias with fair to good reliability^37^, and validity^36^. A rating of the methodological quality of the studies was carried out by both the primary (S.G) and secondary (I.G) reviewer. Ambiguous issues were discussed between the reviewers and a consensus was reached. The interpretation of the rated studies were that studies scoring 9–10 were considered of “excellent”, 6–8 of “good”, 4–5 of “fair”, and <3 of “poor” quality^38^.

### Data Analysis

A within-group i.e. pre-post meta-analysis approach was incorporated in the review to develop a quantitative interpretation of the auditory cueing interventions^39^. The meta-analyses were conducted using CMA (Comprehensive meta-analysis V 2.0, USA). The data in this analysis was distributed and separately analyzed for each outcome measure such as gait velocity, stride length, cadence, and timed-up and go test. Here, the use of either random/fixed effect meta-analysis was dependent upon the presence/absence of heterogeneity during the group analysis, respectively^40^. Moreover, forest plots with 95% confidence intervals were plotted. The effect sizes were adjusted and reported as weighted Hedge’s g^41^. A positive effect size would represent a favorable outcome of the intervention and vice versa for the negative effect. Further, the thresholds for the interpretation of weighted effect sizes are as follows: an effect size of 0.2 is considered as a small effect, 0.5 as a medium effect and 0.8 as a large effect^42^. Further, heterogeneity between the studies was computed using I^2^ statistics^42,43^. The interpretation of heterogeneity via I^2^ statistics is as follows: 0–25% is considered as negligible heterogeneity, 25–75% as moderate heterogeneity and ≥75% as substantial heterogeneity, respectively. In cases where substantial heterogeneity was observed sensitivity analysis were performed to elucidate the “significant” cause of heterogenity^44^. In this analysis, the results were compared by either including or excluding results from studies that used inadequate randomization methods and/or differed in terms of applied intervention.

In the included studies rhythmic auditory cueing was subjected to patients according to their comfortable cadence. The evaluated parameters were spatiotemporal parameters of gait i.e. gait velocity, stride length and cadence. Furthermore, sub-group analyses were also performed to determine specific training dosages for application of rhythmic auditory cueing in a gait rehabilitation protocol. The main emphasis was laid to determine the duration of a training session and the number of days for which these sessions were performed during a week. Likewise, sub-group analyses were also conducted to analyze joint effects of treadmill training together with rhythmic auditory cueing on gait performance in stroke patients. This analysis was performed to analyze the joint influence of adjunct treadmill training with auditory cueing.

Details of weighted effect size, 95% confidence intervals, significance and heterogeneity have been reported for each outcome measure. Additionally, an analysis for publication bias was performed by Duval and Tweedie’s trim and fill procedure^45^. This method involves imputation of the asymmetric studies from the left side to locate the unbiased effect and then re-fills the plot by reinserting the trimmed studies on the left and their imputed counterparts on the right to the mean effect^46^. The graph plots the evaluated weighted effect size i.e. Hedge’s g values against standard error on a random effect model. The alpha level was set at 5%.

## Results

### Characteristics of included studies

**Figure 1:**
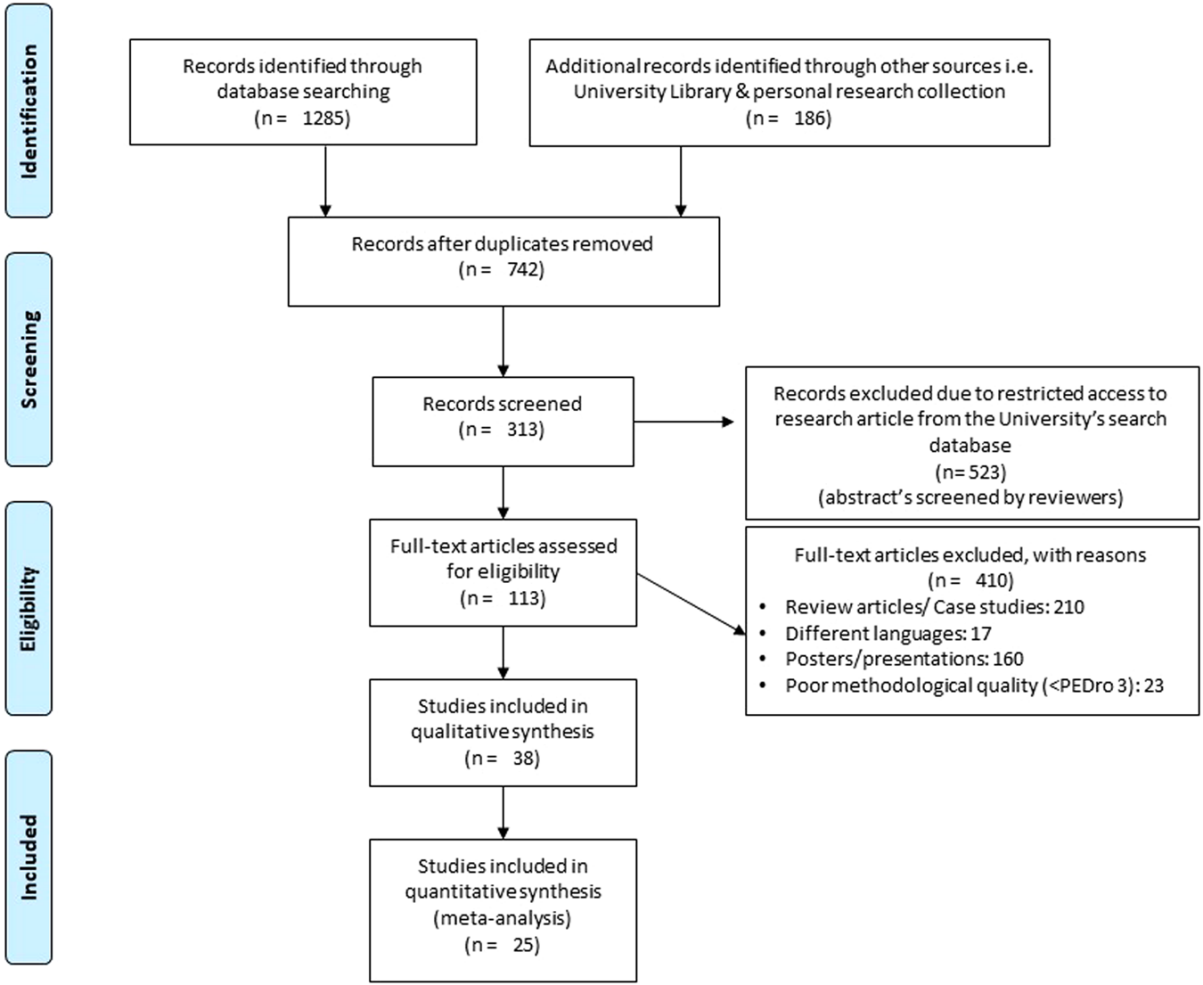
PRISMA flow chart for the inclusion of studies^32^.

The initial search across the academic databases, department’s collection of articles and university’s library repository (additional sources) yielded a total of 1,471 studies, which on implementing our inclusion/exclusion criteria, were reduced to 38 (Fig. 1). Thereafter, quantitative data was extracted from 25 studies. In the remaining studies where quantitative data was either mentioned in figures or not mentioned at all, attempts were made by the reviewers (S.G, I.G) to contact respective authors for relevant data. Qualitative data from the included studies have been summarized in (Supplementary Table 2). Of the 38 included studies, 11 were randomized controlled trials and 27 were controlled clinical trials. All the included studies reported that the stroke patients also received conventional physical therapy in addition to auditory cueing.

### Participants

A total of 968 participants were analyzed in the 38 studies. All the studies included mix gender patients affected from stroke. The included studies provided data on 322 females, and 529 males. Five studies did not specify the gender of the included patients^47–51^. Descriptive statistics relating to the age (mean ± standard deviation) of the participants were tabulated across the studies. Disease duration of stroke patients were also extracted (see Supplementary Table 2), however, five studies did not mention these details^47–51^.

### Risk of bias

**Figure 2:**
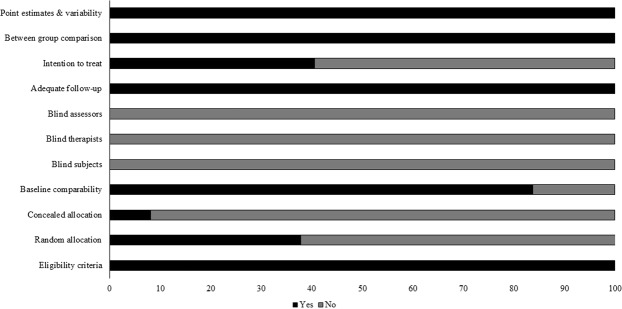
Risk of bias across studies.

Individual scores attained by the studies using the PEDro scale for each factor has been mentioned (Supplementary Tables 1, 2). The average PEDro score for the 38 included studies was computed to be Median (1^st^, 3^rd^ quartile): 5.5 (5, 7) out of 11, indicating on an average a “fair” quality of the studies. During the methodological rating two studies scored eight, nine studies scored seven, nine studies scored six, twelve studies scored five, and six studies scored four (Supplementary Tables 1, 2). Risk of biasing across the studies has also been demonstrated in Fig. 2.

**Figure 3:**
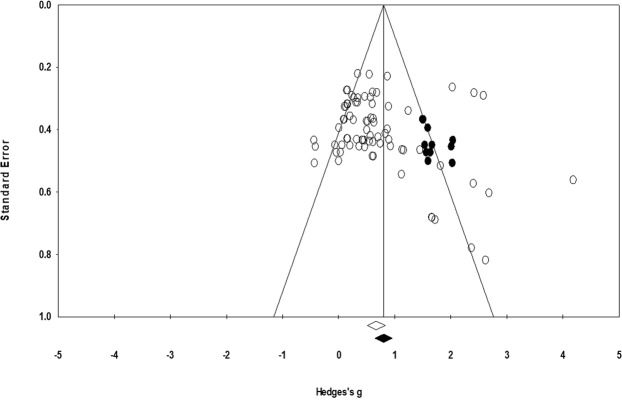
Trim and Fill funnel plot for Hedge’s g and standardized effect for each value in the meta-analysis. Each of the effect is represented in the plot as a circle. Imputed studies are represented by darkened circles. Funnel boundaries represent area where 95% of the effects are expected to lie if there were no publication biases. The vertical line represents the mean standardized effect of zero.

According to the Trim and Fill method 12 studies are missing (Fig. 3). Under the random effects model the point estimate and 95% confidence interval for the combined studies is 0.66 (0.50 to 0.83). Using Trim and Fill method the imputed point estimate is 0.80 (0.64, 0.95).

### Meta-Analysis

#### Outcomes

The current qualitative and quantitative evidence from the review suggests beneficial effects of rhythmic auditory cueing on gait and postural stability performance post-stroke. All 38 studies included in the review reported significant enhancements in gait performance and dynamic postural stability for post-stroke patients with rhythmic auditory cueing (Supplementary Table 2).

### Meta-analysis report

#### Gait velocity

**Figure 4:**
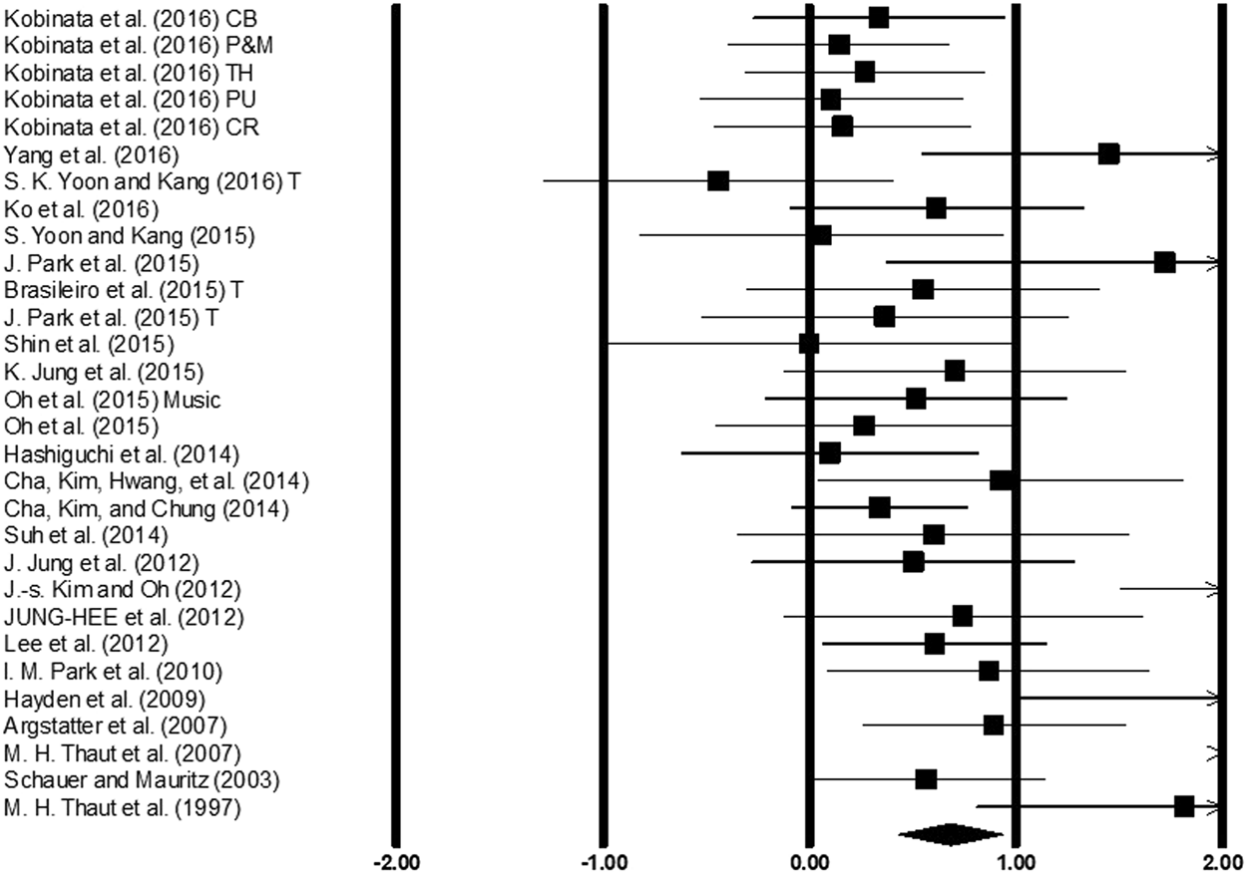
Forest plot illustrating individual studies evaluating the effects of rhythmic auditory cueing on gait velocity amongst post-stroke patients. Weighted effect sizes; Hedge’s g (boxes) and 95% C.I (whiskers) are presented, demonstrating repositioning errors for individual studies. The (Diamond) represents pooled effect sizes and 95% CI. A negative effect size indicated reduction in gait velocity; a positive effect size indicated enhancement in gait velocity. (CB: Cerebellum, P&M: Pons & medulla, TH: Thalamus, PU: Putamen, CR: Corona radiata, T: Treadmill).

Gait velocity was assessed among 25 studies. Additional data concerning different types of auditory stimulations^52^, and lesion sites^53^, in stroke patients was retrieved from two studies (Fig. 4). The analysis of studies revealed (Fig. 4) a medium effect size in the positive domain (g: 0.68, 95% C.I: 0.42 to 0.93) with negligible heterogeneity (I^2^: 7.54%, p > 0.05). Further, a sub-group analysis was performed to evaluate the joint effects of auditory cueing and treadmill gait training (Supplementary Figure 1) among three studies. A small effect size in the positive domain (g: 0.15, 95% C.I: −0.34 to 0.64) was observed with moderate heterogeneity (I^2^: 31.3%, p > 0.05).

Furthermore, we evaluated the effects of training with rhythmic auditory cueing. Based on the current included studies and previous findings^19,27,54^, a training dosage of 20–45 minutes of training session for 3–5 sessions a week was determined. Here, 16 studies with a similar training dosage were included in a sub-group analysis. The analysis of studies revealed (Supplementary Figure 2) a medium effect size in the positive domain (g: 0.73, 95% C.I: 0.39 to 1.08) with no heterogeneity observed in between the studies (I^2^: 0%, p > 0.05). A comparative analysis for a smaller training dosage i.e. 8–10 minutes could not be included in this analysis due to the presence of heterogeneity between the studies. Here, two studies performed gait training with a duration ranging from 8–10 minutes^48,55^. There were differences in between the studies concerning the characteristics of the delivered auditory stimulations. Hayden, *et al*.^55^ for instance, delivered rhythmic auditory cueing according to a patient’s preferred cadence and only allowed increments in tempo ranging from 1–3 bpm. On the contrary, Kim and Oh^48^ subjected their participants to fixed tempo ranging from 20–100 bpm (Supplementary Table 2). Therefore, a comparison of different training dosages was not performed.

Additionally, a comparative sub-group analysis for five studies analyzing effects of rhythmic auditory cueing without training (Supplementary Figure 3) revealed a comparatively smaller medium effect size in the positive domain (g: 0.33, 95% C.I: 0.12 to 0.54) and here as well no heterogeneity was observed in between the studies (I^2^: 0%, p > 0.05).

#### Stride length

**Figure 5:**
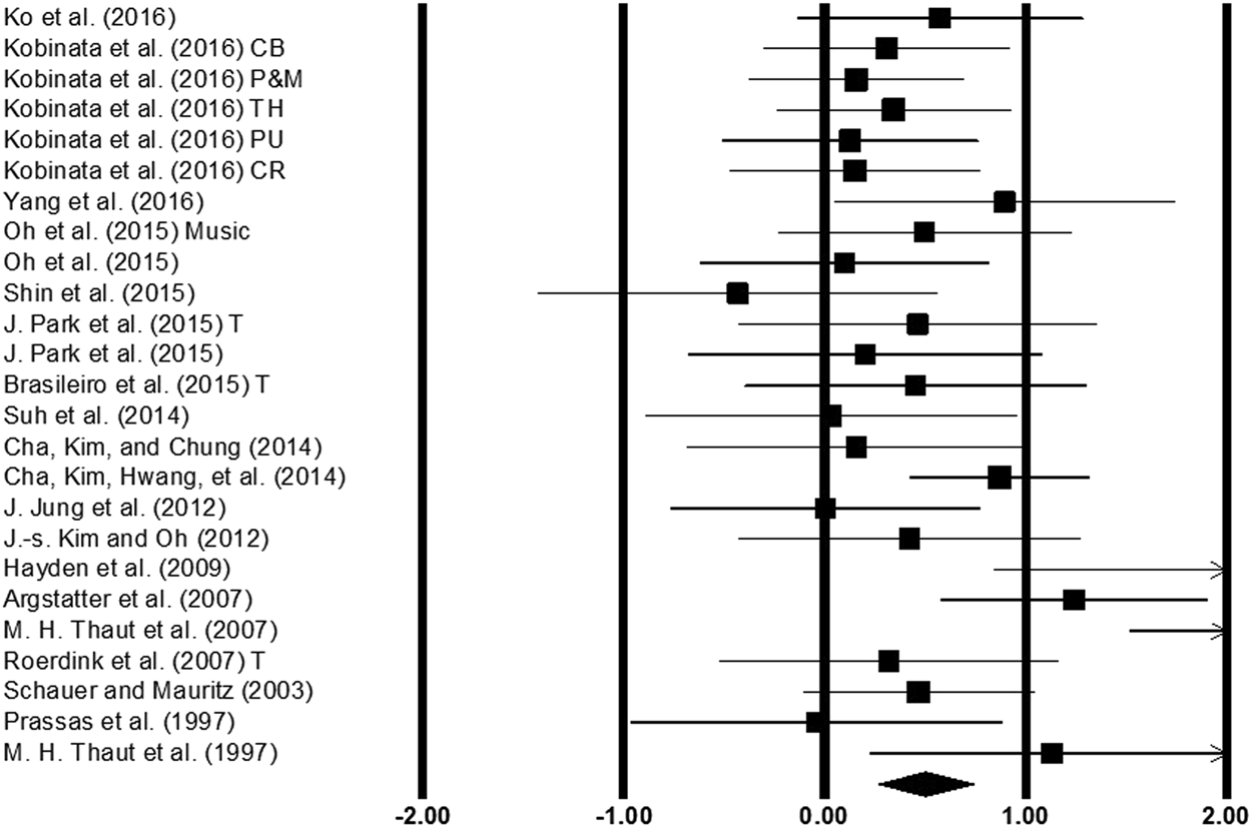
Forest plot illustrating individual studies evaluating the effects of rhythmic auditory cueing, on stride length amongst post-stroke patients. Weighted effect sizes; Hedge’s g (boxes) and 95% C.I (whiskers) are presented, demonstrating repositioning errors for individual studies. The (Diamond) represents pooled effect sizes and 95% CI. A negative effect size indicated reduction in stride length; a positive effect size indicated enhancement in stride length. (CB: Cerebellum, P&M: Pons & medulla, TH: Thalamus, PU: Putamen, CR: Corona radiata, T: Treadmill).

Stride length was assessed among 20 studies. Additional data concerning different: types of auditory stimulations^52^, and lesion sites^53^, in stroke patients was retrieved from two studies. The combined analysis of studies revealed (Fig. 5) a medium effect size in the positive domain (g: 0.50, 95% C.I: 0.26 to 0.73) with no heterogeneity (I^2^: 0%, p > 0.05). Further, a sub-group analysis for two studies evaluated the effects of treadmill gait training with auditory cueing (Supplementary Figure 4). A medium effect size in the positive domain (g: 0.45, 95% C.I: -0.15 to 1.07) was observed with no heterogeneity (I^2^: 0%, p > 0.05).

Also, to determine specific training dosage sub-group analyses were again conducted. Here, 11 studies with a similar training dosage i.e. (20–45 minutes of training session for 3–5 sessions a week) were included in the sub-group analysis. The analysis of studies revealed (Supplementary Figure 5) a medium effect size for this training duration in the positive domain (g: 0.58, 95% C.I: 0.17 to 0.98) and no heterogeneity was observed in between the studies (I^2^: 0%, p > 0.05). Additionally, a comparative sub-group analysis for four studies analyzing effects of rhythmic auditory cueing without training (Supplementary Figure 6) revealed a comparatively smaller medium effect size in the positive domain (g: 0.25, 95% C.I: 0.02 to 0.48) with no heterogeneity (I^2^: 0%, p > 0.05).

#### Cadence

**Figure 6:**
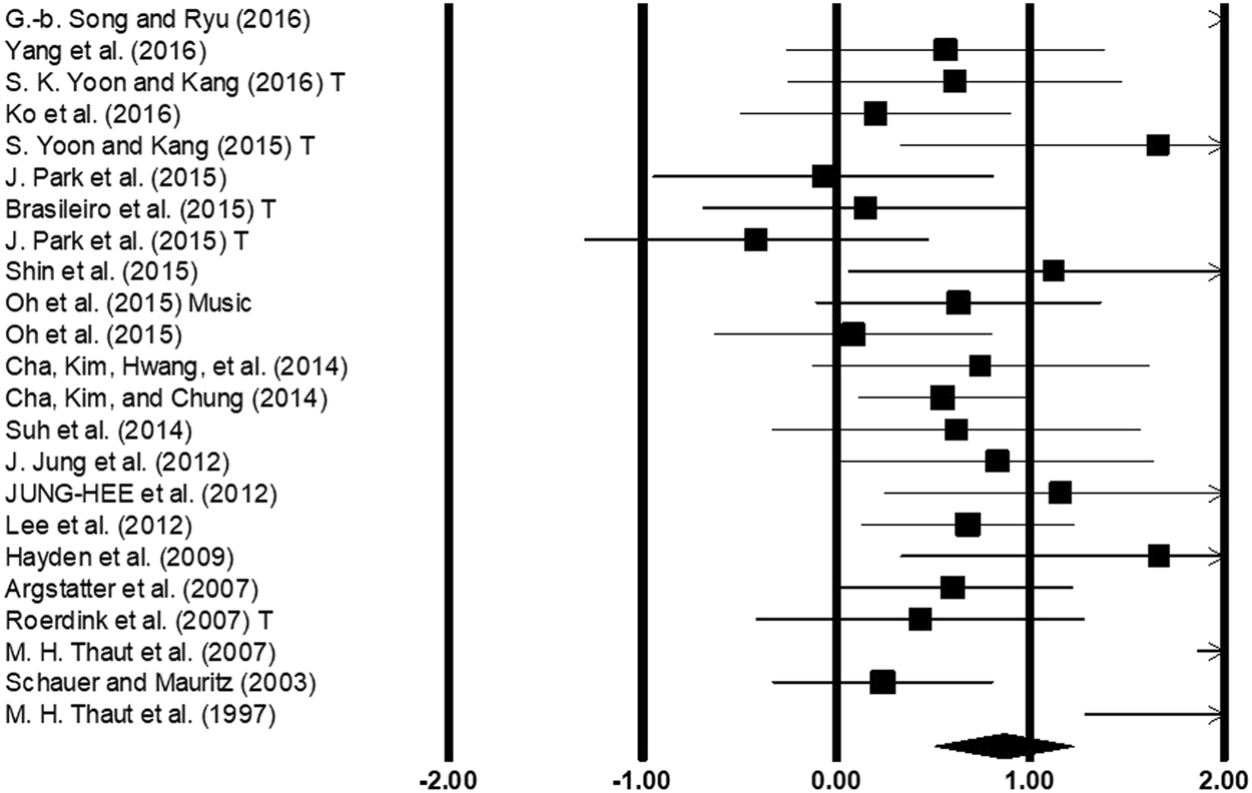
Forest plot illustrating individual studies evaluating the effects of rhythmic auditory cueing, on cadence amongst post stroke patients. Weighted effect sizes; Hedge’s g (boxes) and 95% C.I (whiskers) are presented, demonstrating repositioning errors for individual studies. The (Diamond) represents pooled effect sizes and 95% CI. A negative effect size indicated reduction in cadence; a positive effect size indicated enhancement in cadence. (T: Treadmill).

Cadence was assessed among 23 studies. Additional data was retrieved from one study, concerning a different type of auditory stimulation^52^. The analysis of studies revealed (Fig. 6) a large effect size in the positive domain (g: 0.86, 95% C.I: 0.50 to 1.22) with negligible heterogeneity between the studies (I^2^: 16.7%, p > 0.05). Further, a sub-group analysis for four studies evaluated the effects of treadmill gait training with auditory cueing (Supplementary Figure 7). A medium effect size in the positive domain (g: 0.39, 95% C.I: -0.33 to 1.13) with negligible heterogeneity was observed (I^2^: 14.4%, p > 0.05).

For evaluating effects of specific training dosage further sub-group analyses were conducted. Here, 11 studies with a similar training dosage i.e. (20–45 minutes of training session for 3–5 sessions a week) were included in the sub-group analysis. The analysis of studies revealed (Supplementary Figure 8) a medium effect size in the positive domain (g: 0.75, 95% C.I: 0.34 to 1.10) with moderate heterogeneity (I^2^: 32.8%, p > 0.05). Additionally, a comparative sub-group analysis for four studies analyzing the effects of rhythmic auditory cueing without training (Supplementary Figure 9) revealed a smaller medium effect size in the positive domain (g: 0.52, 95% C.I: 0.17 to 0.87) and no heterogeneity was observed in between the studies (I^2^: 0%, p > 0.05).

#### Timed-up and go test

Time up and go test was assessed among 6 studies. A negative effect size represented enhancement in the performance on timed-up and go test and vice versa for the positive effect size. The analysis of studies revealed (Supplementary Figure 10) a medium effect size in the negative domain (g: −0.76, 95% C.I: −1.36 to −0.16) with moderate heterogeneity in between the studies (I^2^: 25.1%, p > 0.05).

## Discussion

The primary objective of this present systematic review and meta-analysis was to synthesize the current state of knowledge and determine the effects of rhythmic auditory cueing on gait performance and postural stability in stroke patients. The findings from the current meta-analyses suggest positive, *medium*-to-*large* standardized effects (pre vs post intervention effects) of rhythmic auditory cueing to enhance gait performance and dynamic postural stability post-stroke. The main findings are:

1. Spatiotemporal gait parameters were considerably enhanced after training with rhythmic auditory cueing i.e. gait velocity (g: 0.68), stride length (g: 0.50), and cadence (g: 0.86).
2. Dynamic postural stability was considerably enhanced after training with rhythmic auditory cueing i.e. duration of timed-up and go test performance was reduced (g: −0.76).
3. The enhancements in spatiotemporal gait parameters were substantial in studies following a training regime as compared to studies analyzing a direct application of auditory cueing i.e. gait velocity (training: 0.73, no training: 0.33), stride length (training: 0.58, no training: 0.25) and cadence (training 0.75, no training: 0.52).
4. A dose-response analysis revealed that gait and balance training with auditory cueing for 20–45 minutes session, for 3–5 times a week provided maximum increments in spatiotemporal gait and dynamic postural stability performance.

Several reasons can be affirmed to these observed gait and postural performance enhancements after training with auditory cueing. Firstly, from a neurophysiological perspective we presume that auditory cueing could have facilitated the deficit internal neural timing in stroke patients by bypassing the deficit fronto-striatal networks^56^, and the basal ganglia-somatosensory area motor loop^57^, through alternate pathways (see)^58–60^. Moreover, the enhanced sensorimotor synchronization developed between the perception of auditory cueing and gait execution might be due to enhanced periodic/phase corrections^61^. This development of enhanced temporal template/prediction with the auditory stimulations could be due to pre-attentive “micro-timing”, attentive “timescale” processing capabilities of the neural networks mediating phase, periodic corrections, respectively^62^. Secondly, training with auditory cueing could have facilitated re-organization of the deficit neural structures for instance, the stimulation could have increased the motor cortex excitability in the affected hemisphere further resulting in the motor recovery^21,29,63^. Thirdly, based on the findings of Fujioka, *et al*.^64^ we expect that the auditory-motor co-activations could have facilitated neuroplasticity. According to the authors, auditory-motor training could facilitate neuromagnetic β band oscillations (a functional measure representing auditory motor coupling and neuroplasticity^65^) thereby assisting in motor recovery.

In addition to these neurophysiological changes, rhythmic auditory cueing can impart multifaceted effects on musculoskeletal system as well^66–71^. Thaut, *et al*.^72^ suggested that the recruitment and firing rate of motor neurons is determined by the firing rate of auditory neurons (central audiospinal facilitation^73^), which in turn are stimulated with rhythmic entrainment. Likewise, in an electromyographic analysis during gait performance for post-stroke patients, Thaut, *et al*.^74^ revealed that training with auditory cueing reduced muscular co-activation on the paretic side.

Moreover, we observed considerable enhancements in gait performance in studies incorporating training with auditory cueing as compared to direct application of auditory cueing in a single session i.e. gait velocity (training: 0.73, no training: 0.33), stride length (training: 0.58, no training: 0.25) and cadence (training 0.75, no training: 0.52). We presume that these enhancement in performance with training are due to an “entrainment effect” generated as a result of auditory-motor training^68,72^. This effect has been reported to facilitate movement regularity with repetitions (in this context cyclic movements of gait) further resulting in an enhanced “smoothened” learning pattern^26,75–77^. Upon further sub-group analysis we observed differences in terms of performance because of shorter or longer training durations. Here, in a dose-response analysis we observed that a training duration of 20–45 minutes per session provided substantial increments in both the gait and postural performance as compared to shorter training sessions lasting for 8–10 minutes. These dose related findings are in line with a previously published review study reporting beneficial effects of auditory cueing on arm recovery following stroke^16^. Moreover, in light of recent neuroimaging and clinical studies these findings seem plausible^18,24,26^. Bangert and Altenmüller^24^, for instance reported auditory sensorimotor EEG co-activations after only 20 minutes of auditory-motor training. The authors reported this instantaneous plasticity in the cortex with right hemispheric anterior regions, which ideally represent audio-motor integration^24,25^. The authors further added, that this minimum time frame was vital for establishing stimulus response consistency between audio-motor signals. Similarly, Ghai, *et al*.^17^ reaffirmed these findings and revealed enhanced proprioceptive performance^78,79^, after at least 30 minutes of auditory-motor training. According to the authors, this time frame is crucial for establishing an auditory-motor interfaced mapping resulting in a robustly learned skill set^80,81^.

In addition, we would like to point out some important gaps in the current state of literature which could be addressed by future studies. Firstly, importance of home-based interventions has been emphasized in several studies^70,82,83^. Home-based intervention can allow a patient to enhance their performance for daily life activities, and allow a patient to train for a longer duration in a cost-effective manner as compared to in rehabilitation centers^83^. None of the included studies in the current review elucidated the effects of auditory cueing as a home-based intervention. However, in our sub-group analyses we observed that using treadmill (a common home-based exercise modality) together with auditory cueing was an efficient way for enhancing spatiotemporal gait performance in patients with stroke (gait velocity: 0.15, stride length: 0.45, cadence: 0.39). Moreover, recently published review studies have recommended the positive influence of using auditory cueing as a home based intervention to facilitate gait recovery in neurological disorders such as, cerebral palsy^70^, and multiple sclerosis^84^. Therefore, based on the current state of evidence we strongly hypothesize that combining auditory-motor training in both rehabilitation centers and at home will further enhance the prognostic outcome of stroke patients.

Finally, our findings are in line with previously published “high-quality” systematic reviews and meta-analyses reporting *medium*-to-*large* positive effects of training with rhythmic auditory cueing on gait performance in stroke patients. This present study furthers the current state of knowledge concerning the efficacy of auditory cueing intervention for recovering gait, postural performance post-stroke. This review also addresses the limitations of the previously published reviews due to several of the following reasons. Firstly, the present review incorporates a higher number of experimental studies that support our conclusion i.e. 38 studies (968 participants) as compared to previously published reviews including ten (268 participants)^31^, eight (242 participants)^29^, seven (211 participants)^27^, and 2 (40 participants)^29^, studies. This large difference in the number of included studies could be affirmed to a higher number of relevant academic databases searched (with multiple languages) i.e. nine, and the inclusion of controlled clinical trials. Here, the inclusion of controlled clinical trials was justified based on the updated Cochrane guidelines for systematic reviews^85^. The guidelines recommend the addition of controlled clinical trials under the circumstances where data from randomized controlled trials is limited^86^. Secondly, this present review suggests specific training dosages with rhythmic auditory cueing for allowing enhancements in gait performance and postural stability. Thirdly, the present review provides evidence for the beneficial effects of auditory cueing training on dynamic postural stability i.e. timed-up and go test performance. Fourthly, this review provides evidence for the beneficial effects of direct application of rhythmic auditory cueing i.e. no training on gait performance in stroke patients. Lastly, this study provides evidence for the beneficial effects of adjunct training strategies like, treadmill training with rhythmic auditory cueing on gait performance in stroke patients.

Furthermore, we strongly recommend the reader to consider that it is not our intention to disregard the previously published reviews and meta-analyses. These reviews have addressed different factors in stroke recovery (quality of life, arm recovery, cognitive training, gait kinematics, applications by music therapist vs health care practitioner and more), which were not the objectives of the present review. Therefore, in our opinion interpretations should be drawn simultaneously from all the reviews to develop a better understanding of the influence of auditory cueing-based training strategies for stroke recovery.

There are four major limitations in this present review. First, this present systematic review and meta-analysis was not pre-registered in an international prospective register for systematic reviews, such as PROSPERO. Second, lack of descriptive statistics prevented us from including 13 studies in our meta-analysis i.e. out of 38 studies 25 were included. In order to address this limitation multiple attempts were made by the reviewers (S.G and I.G) to retrieve the data from the authors of the respective studies. Thirdly, this meta-analysis evaluated the effectiveness of auditory cueing training from a “pre-post intervention perspective”. This is a major limitation of this study. We refrained from including a comparative analysis with the respective control groups due to limited data for the controlled groups mentioned in the studies. Fourthly, in the present meta-analysis a sensitivity analysis was performed to explore causes of heterogeneity instead of a meta-regression or stratified meta-analysis approach. The choice of this approach could raise concerns regarding the appropriateness to pinpoint the “significant” source of heterogeneity. We justify the choice of sensitivity analysis because it allowed us to simultaneously evaluate three moderators of training i.e. length of training session, number of training sessions per week and number of weeks for which training was performed. This however, was not possible with the use of a conventional meta-regression or stratified meta-analysis approach which only allows the evaluation of a single variable at a given instance.

In conclusion, rhythmic auditory cueing provides beneficial effects for enhancing gait performance and dynamic stability post-stroke. The present findings can be reliably interpreted as limited heterogeneity was ensured during the sub-group analyses, and the included studies had a “fair” overall quality i.e. 5.5. This review strongly suggests the incorporation of rhythmic auditory cueing based training post stroke for enhancing gait performance and postural stability. The review suggests a training duration for at least 20–45 minutes and for at least 3–5 times per week^87–94^.

## Supplementary information

LaTeX Supplementary File
