# Supplementary material for: Effects of (music-based) rhythmic auditory cueing training on gait and posture post-stroke: A systematic review & dose-response meta-analysis
Source: Sci Rep. 2019 Feb 18;9:2183. doi: 10.1038/s41598-019-38723-3 (PMC6379377; doi:10.1038/s41598-019-38723-3)
Supplement: Supplementary file 1 — LaTeX Supplementary File [file 41598_2019_38723_MOESM1_ESM.docx]

Influence of music-based auditory cueing training on gait and balance recovery post stroke: A systematic review & meta-analysis

Supplementary File

Shashank Ghai*, Ishan Ghai

Table 1 Individual Pedro scores for studies (1: point awarded, 0: no point awarded)

| Study | Eligibility criteria | Random allocation | Concealed allocation | Baseline comparability | Blind subjects | Blind therapists | Blind assessors | Adequate follow-up | Intention to treat | Between group comparison | Point estimates & variability | PEDro score |
| --- | --- | --- | --- | --- | --- | --- | --- | --- | --- | --- | --- | --- |
| Kobinata, et al. ^1^ | 1 | 0 | 0 | 1 | 0 | 0 | 0 | 1 | 0 | 1 | 1 | 5 |
| Ko, et al. ^2^ | 1 | 0 | 0 | 1 | 0 | 0 | 0 | 1 | 0 | 1 | 1 | 5 |
| Fouad and Mousa ^3^ | 1 | 0 | 0 | 0 | 0 | 0 | 0 | 1 | 0 | 1 | 1 | 4 |
| Song and Ryu ^4^ | 1 | 0 | 0 | 1 | 0 | 0 | 0 | 1 | 1 | 1 | 1 | 6 |
| Park and Chung ^5^ | 1 | 1 | 1 | 1 | 0 | 0 | 0 | 1 | 1 | 1 | 1 | 8 |
| Yang, et al. ^6^ | 1 | 0 | 0 | 0 | 0 | 0 | 0 | 1 | 0 | 1 | 1 | 4 |
| Yoon and Kang ^7^ | 1 | 1 | 0 | 1 | 0 | 0 | 0 | 1 | 1 | 1 | 1 | 7 |
| Brasileiro, et al. ^8^ | 1 | 0 | 0 | 1 | 0 | 0 | 0 | 1 | 1 | 1 | 1 | 6 |
| Shin, et al. ^9^ | 1 | 0 | 0 | 1 | 0 | 0 | 0 | 1 | 0 | 1 | 1 | 5 |
| Ki, et al. ^10^ | 1 | 0 | 0 | 1 | 0 | 0 | 0 | 1 | 1 | 1 | 1 | 6 |
| Jung, et al. ^11^ | 1 | 0 | 0 | 1 | 0 | 0 | 0 | 1 | 0 | 1 | 1 | 5 |
| Yoon and Kang ^12^ | 1 | 0 | 0 | 1 | 0 | 0 | 0 | 1 | 1 | 1 | 1 | 6 |
| Park, et al. ^13^ | 1 | 0 | 0 | 1 | 0 | 0 | 0 | 1 | 0 | 1 | 1 | 5 |
| Oh, et al. ^14^ | 1 | 1 | 0 | 1 | 0 | 0 | 0 | 1 | 1 | 1 | 1 | 7 |
| Hashiguchi, et al. ^15^ | 1 | 1 | 0 | 1 | 0 | 0 | 0 | 1 | 1 | 1 | 1 | 7 |
| Cha, et al. ^16^ | 1 | 1 | 0 | 1 | 0 | 0 | 0 | 1 | 1 | 1 | 1 | 7 |
| Suh, et al. ^17^ | 1 | 1 | 0 | 1 | 0 | 0 | 0 | 1 | 1 | 1 | 1 | 7 |
| Cha, et al. ^18^ | 1 | 1 | 0 | 1 | 0 | 0 | 0 | 1 | 0 | 1 | 1 | 6 |
| Wright, et al. ^19^ | 1 | 0 | 0 | 0 | 0 | 0 | 0 | 1 | 0 | 1 | 1 | 4 |
| Lee, et al. ^20^ | 1 | 1 | 1 | 1 | 0 | 0 | 0 | 1 | 1 | 1 | 1 | 8 |
| Chouhan and Kumar ^21^ | 1 | 1 | 0 | 1 | 0 | 0 | 0 | 1 | 0 | 1 | 1 | 6 |
| Muto, et al. ^22^ | 1 | 0 | 0 | 1 | 0 | 0 | 0 | 1 | 0 | 1 | 1 | 5 |
| Jung-Hee, et al. ^23^ | 1 | 1 | 0 | 1 | 0 | 0 | 0 | 1 | 1 | 1 | 1 | 7 |
| Kim and Oh ^24^ | 1 | 1 | 0 | 1 | 0 | 0 | 0 | 1 | 1 | 1 | 1 | 7 |
| Jung, et al. ^25^ | 1 | 0 | 1 | 1 | 0 | 0 | 0 | 1 | 0 | 1 | 1 | 6 |
| Johannsen, et al. ^26^ | 1 | 0 | 0 | 1 | 0 | 0 | 0 | 1 | 0 | 1 | 1 | 5 |
| Park, et al. ^27^ | 1 | 0 | 0 | 1 | 0 | 0 | 0 | 1 | 0 | 1 | 1 | 5 |
| Pelton, et al. ^28^ | 1 | 1 | 0 | 1 | 0 | 0 | 0 | 1 | 0 | 1 | 1 | 6 |
| Roerdink, et al. ^29^ | 1 | 1 | 0 | 1 | 0 | 0 | 0 | 1 | 1 | 1 | 1 | 7 |
| Hayden, et al. ^30^ | 1 | 0 | 0 | 1 | 0 | 0 | 0 | 1 | 0 | 1 | 1 | 5 |
| Roerdink, et al. ^31^ | 1 | 0 | 0 | 1 | 0 | 0 | 0 | 1 | 0 | 1 | 1 | 5 |
| Argstatter, et al. ^32^ | 1 | 0 | 0 | 0 | 0 | 0 | 0 | 1 | 0 | 1 | 1 | 4 |
| Thaut, et al. ^33^ | 1 | 1 | 0 | 1 | 0 | 0 | 0 | 1 | 1 | 1 | 1 | 7 |
| Schauer and Mauritz ^34^ | 1 | 0 | 0 | 0 | 0 | 0 | 0 | 1 | 0 | 1 | 1 | 4 |
| Thaut, et al. ^35^ | 1 | 0 | 0 | 0 | 0 | 0 | 0 | 1 | 0 | 1 | 1 | 4 |
| Prassas, et al. ^36^ | 1 | 0 | 0 | 1 | 0 | 0 | 0 | 1 | 0 | 1 | 1 | 5 |
| Thaut, et al. ^37^ | 1 | 0 | 0 | 1 | 0 | 0 | 0 | 1 | 0 | 1 | 1 | 5 |

Table 2 Effects of rhythmic auditory cueing on gait and postural stability in stroke patients

| **Author** | **Research question(s)/ hypothesis** | **Sample description, age: (M ± S.D)** | **PEDro** | **Disease duration** | **Assessment tools** | **Research design** | **Auditory characteristics** | **Conclusion** |
| --- | --- | --- | --- | --- | --- | --- | --- | --- |
| Kobinata, et al. ^1^ | Effects of auditory cueing on gait in patients affected from stroke | Lesion site: Cerebellum 5F, 15M (71.3±9.5)  Pons & medulla: 5F, 21M (67.4±10.9)  Thalamus: 4F, 18M (64±9.2)  Putamen: 7F, 11M (64.3±13.4)  Corona radiata: 7F, 12M (72.8±9.4) | 5 | Cerebellum: 40.8± 30.6 days  Pons & medulla: 38.4± 22.8 days  Thalamus: 61± 33 days  Putamen: 42.7± 19.5 days  Corona radiata: 39.2± 23.2 days | Gait velocity & stride length | Pre-test, gait training with gradually enhanced frequency to achieve increased cadence, rhythm, post-test | Rhythmic metronome cueing (drum or autoharp) at preferred cadence | Significant enhancement in gait velocity, stride length in patients with lesion sites at cerebellum, pons & medulla, thalamus after auditory training.  Enhancement in gait velocity, stride length in patients with lesion sites at putamen, corona radiata after auditory training. |
| Sangita and Remya ^38^ | Effects of auditory cueing on gait in patients affected from stroke | Exp: 15  Ct: 15 | 4 | - | 10-metre walk test, cadence | 3-week training | Rhythmic metronome cueing at preferred cadence | Significant enhancement in 10-metre walk test performance and cadence in Exp as compared to Ct. |
| Ko, et al. ^2^ | Effects of auditory cueing on gait in patients affected from stroke | 4F, 11M (56±7.4) | 5 | 81.9± 87.8 months | Gait speed, cadence, stride length, gait cycle duration, step length affected & unaffected side & symmetry ratio | Pre-test/7 min of gait training, with rhythmic auditory cueing at -10%, -5%, 0%, +5%, +10% of patient’s preferred pace “applied randomly” /post-test | (C-E-G, C-F-A, A-D-G, clap, click, gun & robot sound) at -10%, -5%, 0%, +5%, +10% of patient’s preferred pace | Significant enhancement in cadence, step-length, 10MWT & DGI post training with auditory cueing as compared to Ct group. |
| Fouad and Mousa ^3^ | Effect of rhythmic auditory cueing on treadmill gait in patients affected from stroke | 30 stroke patients  Exp: 15  Ct: 15 | 4 | - | Stride length | Pre-test, treadmill training with (Exp)/without (Ct) rhythmic auditory cueing for 6 weeks, post-test | Rhythmic auditory cueing | Significant enhancement in stride length for both the affected & non-affected side for Exp as compared to Ct. |
| Song and Ryu ^4^ | Effects of auditory cueing on gait in patients affected from stroke | Exp: 8F, 12M (57.1±7.8)  Ct: 11F, 9M (60.1±6.8) | 6 | Exp: 12.3± 3.4 months  Ct: 14.7± 6 months | Cadence, step length, 10 metres walking test & Dynamic gait index | Pre-test, Gait training with/without rhythmic auditory cueing for 30 minutes session, 5 times a week for 4 weeks with rehabilitation/post-test | Rhythmic auditory cueing | Significant enhancement in cadence, step-length, 10MWT & DGI post training with auditory cueing as compared to Ct group. |
| Park and Chung ^5^ | Effects of auditory cueing on robot-assisted gait in patients affected from stroke | Visual cueing: 2F, 3M (52.4±12)  Auditory cueing: 2F, 3M (55±5)  Ct: 3F, 2M (57.2±11.5) | 8 | Visual: 9.2± 1.3 months  Auditory: 9.2± 2.2  Ct: 9.0± 1.5 | Berg balance scale, time-up & go test & 10 metres walking test | Pre-post intervention with robot assisted gait training (40-50% weight supported) for 45 min, 3 times a week for 2 weeks. | Rhythmic auditory cues generated per preferred speed of patients. | Significantly enhanced performance in BBS, TUG, & 10 MWT when participants received auditory cueing as compared to Ct. |
| Yang, et al. ^6^ | Effects of real-time auditory cueing on gait & balance in patients affected from stroke | Exp: 2F, 9M (51.9±13.3)  Ct: 2F, 9M (55.8±13.5) | 4 | Exp: 11.1± 3.6 months  Ct: 11.9± 3.5 months | Gait speed, cadence, step length, stride length, single limb support, gait asymmetry, average perturbation velocity, average total perturbation distance & time up & go test | Pre-test, gait on treadmill training for 30 minutes/ session, 3 sessions/week for 4 weeks with real-time auditory cueing at 0% & 5% input from preferred cadence, post-test | Rhythmic auditory cueing at preferred cadence, tempo modified in two sounds of different pitch, reduced speed by half of averaged gait speed from initial contact of 6th phase of gait cycle at 0% & 5%. | Significant enhancement in gait speed, cadence, step length, stride length, single limb support in Exp as compared to Ct. Significant reduction in gait asymmetry in Exp.  Significant reduction in average perturbation velocity (eye open only), average total permutation distance & time up & go test duration in Exp as compared to Ct with both eyes closed and open performance. |
| Yoon and Kang ^7^ | Effects of auditory cueing on gait performance on treadmill, postural stability in patients affected from stroke | Exp: 4F, 6M (50.8±14.4)  Ct I: 3F, 6M (56.3±7.1)  Ct II: 4F, 5M (61.2±13) | 7 | Exp: 16.4± 10.3 months  Ct I: 13.6± 8.5 months  Ct II: 17.1± 8.4 months | Time up & go test, berg balance score, 6-minute walking test time, gait speed, cadence, single leg stance & symmetry index. | Pre-test, treadmill training at (5% incline, preferred cadence) initially, followed by (10% incline, +5% speed) in 2nd & 3rd weeks, rhythmic auditory cueing for Exp, no auditory cueing for Ct I & Ct II (normal treadmill training), training for 30 minutes’ session, 5 times/ week, for 4 weeks, post-test | Rhythmic metronome cueing at 0% & +5% of preferred cadence | Significant enhancement in berg balance score, gait speed, cadence, single leg stance & symmetry index after training with auditory cueing  Significant reduction in time up & go test, 6-minute walking test time after training with auditory cueing  Significant effects on time up & go test, berg balance score, 6-minute walking test time, gait speed & symmetry index in Exp as compared to Ct I, Ct II. |
| Brasileiro, et al. ^8^ | Effect of auditory cueing on treadmill gait in patients affected from stroke | 12F, 18M  Exp: 10 (58.8±7.9)  Ct: 10 (57.9±4.9)  Ct I: 10 (52.3±5.9) | 6 | Exp: 34.1±20.2 months  Ct I: 37.8± 21.5  Ct: 27.4± 17.4 months | Gait speed, stride length, cadence, paretic stance time, symmetry ratio, maximum hip extension (stance), maximum hip flexion (swing), hip range of motion, knee angle initial contact, maximum knee flexion (swing), knee range of motion, ankle range of motion & ankle angle at initial contact, toe off. | Pre-test, treadmill training with 30% of supported body weight with/without rhythmic auditory cueing at +15% of preferred cadence (Exp), visual cueing (Ct I) for 20 minutes’ session, post-test | Rhythmic auditory cueing at +15% of preferred cadence | Significant enhancement in gait speed, stride length, hip & ankle range of motion after training with rhythmic auditory cueing. No differences between Exp, Ct I & Ct II. |
| Shin, et al. ^9^ | Effects of real-time auditory cueing on gait in patients affected from hemiplegia (stroke/cerebral palsy) | Cerebral palsy: 4F, 3M (30.1±4.1)  Stroke: 4F, 7M (44.2±7) | 5 | Stroke patients: 3.5±2.2 years | Cadence, gait speed, stride length, stride time, step time, single/double support time, stance/swing phase (temporo-spatial deviation & side to side comparison), pelvis, hip, knee, ankle, foot kinematics & gait deviation index | Pre-test, gait training with rhythmic auditory cueing for 30 minutes/ session, 3 sessions/ week for 4 weeks, post-test | Rhythmic auditory cueing by four-chord progression with metronome beat on keyboard | Significantly reduced ankle plantar flexion at initial contact & push off. Reduced anterior pelvic tilt in sagittal plane after training with auditory cueing.  Significantly enhanced kinematic improvements in stroke patients as compared to cerebral palsy.  Significant enhancement in gait deviation index & kinematics for patients affected from sub-acute stroke as compared to chronic stroke.  No effect on gait parameters after training from auditory cueing.  Enhanced side to side symmetry after training from auditory cueing.  Significant enhancement in gait deviation index, hip adduction in mid stance, maximal knee flexion in mid swing, ankle dorsiflexion in terminal stance after training from rhythmic auditory cueing. |
| Ki, et al. ^10^ | Effects of rhythmic auditory on weight bearing phase in gait training and dynamic posture for patients affected from stroke | Exp: 4F, 8M (55.3±9.2)  Ct: 2F, 11M (60.1±12.3) | 7 | Exp: 19.1± 8.2 months  Ct: 22± 9.9 months | Gait parameters (double limb stance, single limb stance phase), time up and go test | Pre (4 weeks training i.e. neurodevelopmental- with/without auditory cueing) post-test analysis | Auditory cueing engaged by pressure gauge when more than 50% weight procured on the healthy. | Significant enhancements in double leg, single leg stance phase and time up & go tests with auditory cueing as compared to control group. |
| Jung, et al. ^11^ | Effect of auditory cueing by cane pressure during gait in patients affected from stroke | Exp: 4F, 7M (56.4±11.1)  Ct: 3F, 7M (56.3±17.1) | 7 | Exp: 6.2± 2.5 months  Ct: 7.0± 2.5 months | Vertical peak force of cane, electromyographip activity of gluteus medius, vastus medialis oblique, single support phase of gait, gait velocity | Pre-test, assisted gait training with/without auditory cueing (calculated by dividing peak vertical force by patients' body weight) with -10% threshold reduction/week, for 30 minutes’ session/day, 5 times/week for 4 weeks, post-test | Real-time auditory cueing at initial threshold of 60% of level of dependency, -10% every week (if comfortable with patient) | Significant enhancement in gait velocity, electromyographic activity of gluteus medius, vastus medialis oblique, single support phase of gait in Exp as compared to Ct.  Significant reduction in vertical peak force of cane in Exp as compared to Ct. |
| Yoon and Kang ^12^ | Effects of auditory cueing on gait performance on treadmill, postural stability in patients affected from stroke | Exp: 3F, 2M (60.6±9)  Ct I: 2F, 3M (57.6±5.5)  Ct II: 2F, 3M (52.8±5.6) | 5 | Exp: 10.4± 2.4 months  Ct I: 9.8± 3.11 months  Ct II: 11.8± 4.3 months | Time up & go test, berg balance score, 6-minute walking test time, gait speed, cadence, single leg stance & symmetry index. | Pre-test, incline treadmill training with rhythmic auditory cueing for Exp, no auditory cueing for Ct I & Ct II (normal treadmill training), training for 30 minutes’ session, 5 times/ week, for 3 weeks, post-test | Rhythmic metronome cueing at preferred cadence | Significant enhancement in berg balance score, gait speed, cadence, single leg stance & symmetry index after training with auditory cueing  Significant reduction in time up & go test, 6-minute walking test time after training with auditory cueing |
| Park, et al. ^13^ | Effect of rhythmic auditory cueing & treadmill training on gait in patients affected from stroke | Exp I: 5F, 4M (51.8±12.5)  Exp II: 4F, 6M (55±9.8) | 6 | Exp I: 10.3± 3.3 months  Exp II: 12.5± 4.2 months | Gait speed, step cycle, step length (affected/unaffected side), coefficient of variation of gait cycle (affected/unaffected side), functional gait assessment, 6-minute walking distance test & timed up & go test | Pre-test, gait training with treadmill (Exp I), normal ground walking (Exp II) with rhythmic auditory cueing progressing at -10% (1st week), 0% (2nd week), +10% (3rd week) of preferred cadence, for 30 minutes’ session, 5 times/week for 3 weeks, post-test | Rhythmic metronome cueing at -10%, 0%, +10% of preferred cadence | Significant reduction in coefficient of variation of gait cycle (affected/unaffected side), step cycle in Exp I & Exp II.  Significant enhancement in functional gait assessment, 6-minute walking distance test, gait speed, step length (affected/unaffected side) in Exp I & Exp II.  Reduction in time up & go test time in Exp I & Exp II. |
| Oh, et al. ^14^ | Effects of auditory cueing on gait, postural stability in patients affected from stroke | Exp I: 8F, 6M (55.8±8)  Exp II: 7F, 7M (57.4±8) | 5 | Exp I: 8.3± 2.3 months  Exp II: 8.9± 1.9 months | Gait velocity, cadence, stride length, double limb support, time up & go test, functional gait assessment & centre of body sway angle (x, y, z axis) | Pre-test, gait training with rhythmic auditory cueing (Exp I: music, Exp II: metronome) at preferred cadence for first week followed by +10% for the second & third week, training for 30 minutes’ session, 5 times/ week, for 3 weeks, post-test | Exp I: Rhythmic auditory cueing on music (2/4 & 4/4-time signature)  Exp II: Rhythmic metronome cueing | Significant enhancement in Gait velocity, cadence, stride length, functional gait assessment (music>metronome) after training with auditory cueing.  Significant reduction in time up & go test (music> metronome), centre of body sway, double limb support after training with auditory cueing |
| Hashiguchi, et al. ^15^ | Effect of rhythmic auditory cueing on gait & muscle activity in patients affected from stroke | 14 patients | 4 | - | Gait velocity, coefficient of variation for stride time, coefficient of variation of duration time, electromyographic activity of gastrocnemius & tibialis anterior | Pre-test, gait performance with rhythmic auditory cueing at 0%, +10% of preferred cadence, adjusted for stride-to-stride tempo for paretic/non-paretic limb, post-test | Rhythmic auditory cueing at 0%, +10% of preferred cadence, adjusted for stride-to-stride tempo for paretic/non-paretic limb | Significant enhancement in gait velocity, electromyographic activity of gastrocnemius with rhythmic auditory cueing at +10% of preferred cadence as compared to baseline.  Significant reduction in coefficient of variation of stride time, coefficient of variation of duration time with rhythmic auditory cueing at +10% of preferred cadence as compared to baseline. |
| Cha, et al. ^16^ | Effect of auditory cueing on gait in patients affected from stroke | 17F, 24M (60.8±19.8) | 7 | 8.68± 2.35 months | Patients walked at preferred speed followed by rhythmic auditory cueing applied randomly at -10%, 0%, +10%, +20% of basic tempo while performing gait. | Gait velocity, cadence, stride length, double limb support, double single limb support  Gait symmetry ratio | -10%, 0%, +10%, +20% of basic tempo for metronome adjusted at patients preferred pace. | Significantly reduced gait velocity, cadence & stride length with -10% of rhythmic auditory stimuli as compared to 0%  Significant enhancement of gait symmetry with normalized auditory stimulus.  Significant enhancement in gait velocity & cadence in +10% & +20% auditory stimuli. However reduced gait symmetry as compared to 0% condition. |
| Suh, et al. ^17^ | Effect of auditory cueing on gait & balance in patients affected from stroke | Exp: 5F, 3M (61±14.4)  Ct: 5F, 3M (70.6±12.4) | 6 | Exp: 386.3± 283.2 days  Ct: 224.2± 213 days | Cadence, gait velocity, stride length, overall stability index & anterior-posterior, mediolateral stability index | Pre-test, gait training with rhythmic auditory cueing at 0%, +5%, +10% of preferred cadence for 30 minutes/day, 5 times a week for 3 weeks, post-test | Rhythmic tone cueing, with single tone series in 4/4-time signature, 60dB, 40-100bpm, at 0%, +5% & +10% of preferred cadence | Significant enhancement in gait velocity, overall stability index & anterior-posterior, mediolateral stability index after training in Exp as compared to before training & Ct.  Enhancement in cadenceafter training in Exp as compared to before training & Ct.  No effect on stride length. |
| Cha, et al. ^18^ | Effects of rhythmic auditory cueing on gait & posture in patients affected from stroke | Exp: 4F, 6M (59.8±11.7)  Ct: 4F, 6M (631±4.1) | 7 | Exp: 14.5± 5.5  Ct: 14.7± 5.4 | Berg balance scale, gait velocity, cadence, stride length (affected/unaffected side), double stance period (affected/unaffected side), stroke specific quality of life scale | Pre-test, gait training with rhythmic auditory cueing at 0% of preferred cadence for 30 minutes/session, 5 times/week, for 6 weeks (+5% of preferred cadence on 3rd & 5th week), post-test | Rhythmic auditory cueing, metronome superimposed on music at 0%, +5% of preferred cadence | Significant enhancement in berg balance score, gait velocity, cadence, stride length (affected/unaffected side), stroke specific quality of life scale after training with rhythmic auditory cueing, in Exp as compared to Ct.  Significant reduction in double stance period (affected/unaffected side) after training with rhythmic auditory cueing, in Exp as compared to Ct. |
| Wright, et al. ^19^ | Effect of rhythmic auditory cueing on gait in patients affected from stroke | 4F, 6M (61±16) | 6 | 6± 2 years | Step time asymmetry, paretic step time variability, nonparetic step time variability & time up & go test | Pre-test, gait performance with/without rhythmic auditory cueing of single & dual tones (randomized) | Rhythmic metronome cueing at single tone (700Hz)  Rhythmic metronome cueing at dual tone (700Hz & 1400Hz) | Significant reduction in step time asymmetry (single tone only) & paretic step time variability with both single & dual tone rhythmic auditory cueing.  Reduction in non-paretic step time variability with both single & dual tone rhythmic auditory cueing. |
| Lee, et al. ^20^ | Effect of auditory cueing on gait & in patients affected from stroke | 11F, 14M (64.3±8.2) | 8 | 12.8± 7.5 months | Gait velocity, cadence, symmetry index, symmetry ratio & gait asymmetry | Gait performance with rhythmic auditory cueing at preferred cadence, paretic/non-paretic leg footfall with auditory cueing at preferred cadence, ±30% of preferred cadence | Rhythmic metronome cueing at 0% & ±30% of preferred cadence | Significant enhancement in gait velocity, symmetry & cadence when auditory cueing was directed at paretic limb at 0% & ±30% of preferred cadence. |
| Chouhan and Kumar ^21^ | Effect of rhythmic auditory cueing on gait & arm reaching in patients affected from stroke | Exp: 3F, 12M (56.7±5.9)  Ct I: 3F, 12M (58.1±4.1)  Ct II: 3F, 12M (57.3±5.5) | 6 | - | Dynamic gait index & Fugyl meyer motor scale score | Pre-test, gait, reaching task training with rhythmic auditory cueing (0% of preferred cadence initially, increased by +10% every week if comfortable for patient: for gait) (Exp) or visual cueing (Ct I) for 2 hours training, 3 time/week session for 3 weeks, post-tests at 7, 14, 21, 28 days | Rhythmic auditory cueing at 0% & +10% on following weeks of preferred cadence | Significant enhancements in dynamic gait index & Fugyl meyer motor scale (14, 21, 28 days only) after 7, 14, 21, 28 days of training with rhythmic auditory cueing & in Exp as compared to Ct II. |
| Muto, et al. ^22^ | Effect of rhythmic auditory cueing in gait for patients affected from stroke | Exp: 3F, 5M (57.5±12.6)  Ct: 3F, 5M (57.1±15.6) | 5 | Exp: 11.8± 14.3 months  Ct: 15.1± 18.8 months | Left-right phase difference (gait asymmetry), (fluctuation in gait tempo) standard deviation of ground contact period during leg motion | Gait training for 9 sessions with rhythmic auditory cueing at +5% of preferred cadence(Ct), walk-mate (rhythmic real-time auditory cueing) (Ct), pre-test & post-tests at the beginning & end of 9 sessions | Walkmate auditory cueing (real-time): Continuous rhythmic auditory cueing according to gait pattern  Stable phase difference computed with gait pattern  Internal model modulates frequency by target phase difference to adapt to changing gait pattern  Rhythmic auditory cueing (dual-dynamics model) +5% of preferred cadence | Significant reduction gait asymmetry in Exp during training with walk-mate auditory cueing, improvements not retained after training.  No effect on gait asymmetry with rhythmic auditory cueing at +5% of preferred cadence.  Significant reduction in fluctuation in gait tempo for for Exp during>after walk-mate auditory training.  Significant reduction in fluctuation in gait tempo for Ct during gait training with rhythmic auditory cueing at +5% of preferred cadence. |
| Jung-Hee, et al. ^23^ | Effect of auditory cueing on gait & postural stability in patients affected from stroke | Exp: 4F, 6M (58.3±11.8)  Ct: 3F, 7M (51.8±13.7) | 7 | Exp: 5.68± 1.04 months  Ct: 4.76± 2.65 | Activities specific balance confidence scale, dynamic gait index, four square step tests, functional ambulation category score, timed up & go test, stair up & down steps/sec gait velocity, stride length, gait cycle time & cadence | Pre-test, functional gait training with rhythmic auditory cueing for 30 minutes training session, 3 times per week for 5 weeks, post-test | Rhythmic metronome cueing at +5% for normal preferred cadence (-20% when gait was unmatched with given rhythmic auditory cueing) | Significant enhancement in gait velocity, activities specific balance confidence scale, dynamic gait index, cadence, functional ambulation category score, stride length (affected & unaffected side) after training with auditory cueing.  Significant reduction in gait cycle time on unaffected side, four square step test, time up & go test, stair up & down steps/sec after training with auditory cueing.  Significantly enhanced performance in activities specific balance confidence scale, dynamic gait index & timed up & go test in Exp as compared to Ct.  Reduction in gait cycle time on affected side after training with auditory cueing. |
| Kim and Oh ^24^ | Effect of rhythmic auditory cueing on gait in patients affected from stroke | Exp: 10 (65.2±6.8)  Ct: 10 (64.5±8.1) | 7 | Exp: 15.2± 2.3 months  Ct: 15.3± 3 months | Stride length (affected/unaffected side), stride length ratio, support time (affected/unaffected side), single support time ratio & gait velocity | Pre-test, gait training for 10 minutes’ session, 3 times/week for 6 weeks with rhythmic auditory cueing at 20, 40, 60, 80, & 100 bpm incremented at 0, 2, 4, 6 & 8 minutes of training, post-test | Rhythmic metronome cueing at 20, 40, 60, 80, & 100 bpm | Significant enhancement in stride length (affected/unaffected side), support time (affected/unaffected side) & gait velocity in Exp as compared to Ct.  Significant reduction in single support time ratio & stride length ratio in Exp as compared to Ct. |
| Jung, et al. ^25^ | Effect of rhythmic auditory cueing on gait in patients affected from stroke | 5F, 7M (52.5±12.4) | 6 | 15.5± 8.5 months | Gait velocity, cadence, stride length & step length | Gait performance with visual & rhythmic auditory cueing at 0%, ±50% of preferred cadence | Rhythmic auditory cueing at 0%, ±50% of preferred cadence | Significant effect of combined visual-auditory cueing on gait velocity i.e. reduced gait parameters with reduced cueing (-50% cueing of preferred cadence) & vice versa for enhanced cueing (+50% cueing of preferred cadence) |
| Johannsen, et al. ^26^ | Effect of rhythmic auditory cueing on arm reaching & gait in patients affected from stroke | Exp I: 3F, 8M (59.513.4)  Exp II: 3F, 7M (68.110.1) | 7 | 62.5± 50.9 months | Fugyl meyer motor assessment (upper/lower extremity), 10-meter walking test, treadmill (step length), repetitive foot/hand aiming task | Pre-test, bilateral (arm: Exp I/leg: Exp II) training with rhythmic auditory cueing for 45 minutes’ session, 2 times/week for 5 weeks, post-test, follow up post-test after 18 weeks | Rhythmic auditory cueing at preferred pace of physical activity (increased at patient’s preference)  bilateral leg training with rhythmic auditory cueing: increased during training from 36.7±6.5-45.9±9.5  bilateral arm training with rhythmic auditory cueing: increased during training from 39.8±5.6-46.3±5.9 | Significant enhancement in treadmill step length on both paretic & non-paretic side after bilateral leg training in Exp II as compared to Exp I (no effects), during immediate follow-up test. No effects in follow up post-test.  Enhancement in fugl meyer motor test for lower extremity in Exp II> Exp I at post-test. No enhancements in follow up post-test  Enhancement in fugl meyer motor test for upper extremity in Exp I> Exp II at post-test. No enhancements in follow up post-test  Enhancement in treadmill step length on both paretic & non-paretic side after bilateral arm training in Exp I as compared to Exp II during 18 week follow up post-test.  Enhancement in repetitive foot & arm aiming task on both paretic & non-paretic side after bilateral leg training in Exp II during immediate post-tests. No effects on follow up post-tests. |
| Park, et al. ^27^ | Effects of auditory cueing on gait in patients affected from stroke | Exp: 5F, 8M (59.2±11)  Ct: 4F, 8M (52.9±13) | 7 | Exp: 15.5± 5 months  Ct: 14± 8 months | Gait speed, number of steps & Wisconsin gait scale | Pre-test, gait training with rhythmic auditory cueing at 30 minutes’ session, twice a day, 5 days/week, for 2 weeks, post-test | Rhythmic auditory cueing (120 bpm) embedded in music | Significant enhancement in gait speed in Exp as compared to Ct.  Significant reduction in number of steps & Wisconsin gait scale in Exp as compared to Ct. |
| Pelton, et al. ^28^ | Effects of auditory cueing on treadmill gait in patients affected from stroke | 3F, 5M (70±12) | 5 | 41.5± 32.2 months | Baseline asynchrony, percentage proportional error in period control, limb symmetry, correction parameter & relative asymptope | Gait performance with 20 metronome pulses without phase shift, followed by 80 pulses with random 1 phase shift (counterbalanced for paretic & non-paretic limb) i.e. delayed metronome cueing | Rhythmic metronome cueing, 1 phase shift: 20% of inter pulse interval i.e. 36º of gait cycle | Significant reduced correction for phase shifts when error occurred on nonparetic limb (correction required on paretic side) as compared to paretic limb, vice versa with rhythmic auditory cueing (with phase shifts) |
| Roerdink, et al. ^29^ | Effects of auditory stimuli on gait performance in patients affected from stroke | Exp: 4F, 7M 60 (42-71)  Ct (healthy): 4F, 6M 60(46-79) | 5 | Exp: 18.5± 17.5 months | Mean phase relation between footfall & acoustic stimuli, step width, spatial-temporal gait asymmetry, variability of relative timing between footfall & metronome beat | Patients performed gait with/without auditory pacing input for single (paretic/non-paretic limb), double (both limbs) metronome, thereafter gait performed and auditory input delivered off-time & patients synchronized with tone. | Single & double paced rhythmic auditory cueing, sampled at 1000Hz. | Significantly enhanced auditory-motor synchronization in condition of double as compared to single-metronome condition.  Patients had slower step response to restore synchronization when auditory stimuli were presented later as compared to before. Ct group had better & faster step response as compared to Exp.  Step width increased with acoustic pacing for both Exp & Ct. |
| Hayden, et al. ^30^ | Effects of auditory cueing on gait & postural stability in patients affected from stroke | Exp I: 1F, 4M (55-72 years)  Exp II: 4F, 1M (55-72 years)  Exp III: 3F, 2M (55-72 years) | 5 | - | One limb stance, cadence, gait velocity, stride length, timed up & go test, functional reach test & postural changes by head tilt measurement | Pre-test, Gait training for (Exp I: 30 sessions with auditory cueing, Exp II: 20 sessions with auditory cueing, Exp III: 10 sessions with auditory cueing) 8-10minutes day 1, after 10 sessions, after 20 sessions, post-tests at 1st, 11th, 21st & 30th session | Rhythmic auditory “music” cueing at preferred cadence & increased by 1-3 bpm (when patient comfortable) | Significant improvements for the timed up and go test and the functional reach test.  Significantly enhanced one-limb stance and cadence with earlier implementations of rhythmic auditory cueing in treatment protocol |
| Roerdink, et al. ^31^ | Effect of rhythmic auditory cueing on treadmill gait in patients affected from stroke | Exp: 2F, 8M (63, 46-78)  Ct (healthy): 5F, 4M (69, 60-78) | 5 | Exp: 37.7± 32.6 months | Stride frequency, stride length, step length (paretic, nonparetic side), spatial asymmetry, stride time, step time (paretic, nonparetic side), step width, interlimb coordination (relative phase difference, relative phase variability) | Gait performance on treadmill with (Exp)/without (Ct) with rhythmic auditory cueing at 0%, ±10% of preferred cadence | Rhythmic auditory cueing (0%, ±10% of preferred cadence) on alternate left & right ear | Significant effect of rhythmic auditory cueing on stride frequency (enhanced: +10%, reduced: -10%), stride length (reduced: +10%, enhanced: -10%), step length (paretic, non-paretic side: reduced: +10%, enhanced: -10%), stride time (reduced: +10%, enhanced: -10%), step time (paretic, non-paretic side: reduced: +10%, enhanced: -10%) & step width (reduced: +10%, enhanced: -10%)with rhythmic auditory cueing at 0% & 10% of preferred cadence for Exp.  Significant enhancement in relative phase difference with pacing stimuli for Exp with rhythmic auditory cueing.  Significant reduction in spatial asymmetry, temporal asymmetry with pacing rhythmic auditory cueing for Exp. |
| Argstatter, et al. ^32^ | Effects of rhythmic auditory cueing on gait in patients affected from stroke | Exp: 9F, 11M (69.3±10.2)  Ct: 8F, 12M (69.2±9.5) | 4 | Exp: 20.7±0.2 days  Ct: 24.2± 5.3 days | Gait velocity, stride length, cadence, gait cycle, gait symmetry, Barthel index, Fugl meyer motor assessment, functional independence measure | Pre-test, gait training with (Exp)/without (Ct) rhythmic auditory cueing at preferred cadence for a 30 minutes’ session/day for 3 weeks, post-test | Rhythmic auditory cueing at preferred cadence (autoharp) with tempo changed according to patient’s performance (2/4 pattern) | Significant enhancement in barthel index score, functional independence measure (no difference between Exp & Ct) in Exp after training with rhythmic auditory cueing & as compared to Ct.  Significant enhancement in gait velocity, cadence, stride length in Exp after training with rhythmic auditory cueing, no difference with Ct.  Enhancement in Fugyl meyer motor test, gait symmetry in Exp after training with rhythmic auditory cueing.  Reduction in gait cycle in Exp after training with rhythmic auditory cueing. |
| Thaut, et al. ^33^ | Effects of auditory stimuli on gait performance in patients affected from stroke | Exp: 21F, 22M (69.2±11)  Ct: 16F, 19M (69.7±11) | 7 | Exp: 21.3± 11 days  Ct: 22.2± 12 days | Gait velocity, stride length, cadence, symmetry (swing ratio) | Pre-test, training with repeated auditory input for Exp & neurodevelopmental therapy/Bobath therapy for Ct for 30 min/5 times a week for 3 weeks, test after 3 weeks, 6-week post-test.  Exp auditory input: Phases: 1^st^: preferred pace, 2^nd^: +5%, 3^rd^: ramp & step training, 4^th^ fading auditory input. | Metronome input at preferred pace, +5%. | Significant enhancement in Gait velocity, stride length, cadence, symmetry as compared to Ct after 3 & 6 week training. |
| Schauer and Mauritz ^34^ | Effects of auditory cueing on gait in patients affected from stroke | 23 patients  Exp: (59±12)  Ct: (61±12) | 4 | Exp: 53 days  Ct: 67 days | Gait velocity, stride length, cadence, symmetry deviation, stride frequency & heel on-toe-off distance | Pre-test, gait training with music motor cueing for 20 min session, 5 days/week, 15 total sessions. | Music motor cueing adjusted for preferred cadence by time interval adjusted between consecutive heel strikes | Significant enhancement in gait velocity, stride length, heel on-toe-off distance.  Significantly reduced symmetry deviation.  Enhanced cadence with auditory cueing. |
| Thaut, et al. ^35^ | Effects of auditory cueing on gait & muscle activity in patients affected from stroke | Exp: 5F, 5M (73±7)  Ct: 5F, 5M (72±8) | 4 | - | Gait velocity, stride length, gait symmetry cadence, Electromyogram amplitude variability (Gastrocnemius) | Pre-test/ training for 60 minutes with rhythmic auditory input/ post-tests  Increased rhythmic auditory cueing by +5%, +10% of preferred cadence in the later stage of training. | Rhythmic metronome cueing superimposed on music for rhythmic input at 0%, +5%, +10% of preferred cadence, subdivided basic meter in ratios 1:2, 1:4. | Significant enhancement in gait velocity, stride length gait symmetry & cadence in Exp.  Significant reduction in electromyogram amplitude variability of gastrocnemius in Exp. |
| Prassas, et al. ^36^ | Effects of auditory cueing on gait & muscle activity in patients affected from stroke | 1F, 7M (69.6±11) | 5 | 7.75± 7.24 months | Stride length, knee, hip joint range of motion, trunk angle, pelvic tilt, centre of mass displacement for vertical & lateral mass, centre of mass horizontal velocity & Electromyogram amplitude variability (Gastrocnemius) | Gait performance tested with/without rhythmic auditory cueing | Rhythmic auditory cueing at preferred cadence (original music composition allowed accentuation of 1^st^ & 3^rd^ beats) | Significant enhancement in stride length symmetry & symmetry of hip joint range of motion on both affected & non-affected side with rhythmic auditory cueing  Significant reduction in centre of mass vertical displacement with rhythmic auditory cueing |
| Thaut, et al. ^37^ | Effects of auditory cueing on gait & muscle activity in patients affected from stroke | 2F, 8M (70.4±10.4) | 5 | 6.5± 6.91 months | Stride variation, symmetry, weight bearing during stance, Electromyogram amplitude variability (Gastrocnemius) | Gait performance tested with/without rhythmic auditory cueing 3 times for 5 weeks | Rhythmic auditory cueing at 4/4-time signature (1^st^ & 3^rd^ beat accentuated by tambourine beat) at preferred cadence | Significant enhancement in weight bearing stance time on affected side & stride symmetry when rhythmic auditory cueing was received.  Significant enhancement of magnitude of muscle activation during midstance/push-off on affected side & reduced on un-affected side.  Significant reduction in electromyographic variability during swing phase on affected side (correlated with enhancement in stride symmetry).  Significant reduction in variability of integrated amplitude ratios during midstance/push-off phase on affected side. |

Figure 1 Forest plot illustrating individual studies evaluating the effects of rhythmic auditory cueing on gait velocity amongst post stroke patients with treadmill. Weighted effect sizes; Hedge’s g (boxes) and 95% C.I (whiskers) are presented, demonstrating repositioning errors for individual studies. The (Diamond) represents pooled effect sizes and 95% CI. A negative effect size indicated reduction in gait velocity; a positive effect size indicated enhancement in gait velocity. (T: Treadmill)

Figure 2 Forest plot illustrating individual studies evaluating the effects of rhythmic auditory cueing with training on gait velocity amongst post-stroke patients. Weighted effect sizes; Hedge’s g (boxes) and 95% C.I (whiskers) are presented, demonstrating repositioning errors for individual studies. The (Diamond) represents pooled effect sizes and 95% CI. A negative effect size indicated reduction in gait velocity; a positive effect size indicated enhancement in gait velocity. (CB: Cerebellum, P&M: Pons & medulla, TH: Thalamus, PU: Putamen, CR: Corona radiata, T: Treadmill)

Figure 3 Forest plot illustrating individual studies evaluating the effects of rhythmic auditory cueing with no training on gait velocity amongst post-stroke patients. Weighted effect sizes; Hedge’s g (boxes) and 95% C.I (whiskers) are presented, demonstrating repositioning errors for individual studies. The (Diamond) represents pooled effect sizes and 95% CI. A negative effect size indicated reduction in gait velocity; a positive effect size indicated enhancement in gait velocity. (CB: Cerebellum, P&M: Pons & medulla, TH: Thalamus, PU: Putamen, CR: Corona radiata, T: Treadmill)

Figure 4 Forest plot illustrating individual studies evaluating the effects of rhythmic auditory cueing with treadmill on stride length amongst post-stroke patients with treadmill. Weighted effect sizes; Hedge’s g (boxes) and 95% C.I (whiskers) are presented, demonstrating repositioning errors for individual studies. The (Diamond) represents pooled effect sizes and 95% CI. A negative effect size indicated reduction in stride length; a positive effect size indicated enhancement in stride length. (T: Treadmill)

Figure 5 Forest plot illustrating individual studies evaluating the effects of rhythmic auditory cueing with training on stride length amongst post-stroke patients. Weighted effect sizes; Hedge’s g (boxes) and 95% C.I (whiskers) are presented, demonstrating repositioning errors for individual studies. The (Diamond) represents pooled effect sizes and 95% CI. A negative effect size indicated reduction in stride length; a positive effect size indicated enhancement in stride length. (CB: Cerebellum, P&M: Pons & medulla, TH: Thalamus, PU: Putamen, CR: Corona radiata, T: Treadmill)

Figure 6 Forest plot illustrating individual studies evaluating the effects of rhythmic auditory cueing with no training on stride length amongst post-stroke patients. Weighted effect sizes; Hedge’s g (boxes) and 95% C.I (whiskers) are presented, demonstrating repositioning errors for individual studies. The (Diamond) represents pooled effect sizes and 95% CI. A negative effect size indicated reduction in stride length; a positive effect size indicated enhancement in stride length. (CB: Cerebellum, P&M: Pons & medulla, TH: Thalamus, PU: Putamen, CR: Corona radiata, T: Treadmill)

Figure 7 Forest plot illustrating individual studies evaluating the effects of rhythmic auditory cueing, on cadence amongst post stroke patients with treadmill. Weighted effect sizes; Hedge’s g (boxes) and 95% C.I (whiskers) are presented, demonstrating repositioning errors for individual studies. The (Diamond) represents pooled effect sizes and 95% CI. A negative effect size indicated reduction in cadence; a positive effect size indicated enhancement in cadence. (T: Treadmill)

Figure 8 Forest plot illustrating individual studies evaluating the effects of rhythmic auditory cueing with training on cadence amongst post-stroke patients. Weighted effect sizes; Hedge’s g (boxes) and 95% C.I (whiskers) are presented, demonstrating repositioning errors for individual studies. The (Diamond) represents pooled effect sizes and 95% CI. A negative effect size indicated reduction in cadence; a positive effect size indicated enhancement in cadence. (T: Treadmill)

Figure 9 Forest plot illustrating individual studies evaluating the effects of rhythmic auditory cueing with no training on cadence amongst post-stroke patients. Weighted effect sizes; Hedge’s g (boxes) and 95% C.I (whiskers) are presented. The (Diamond) represents pooled effect sizes and 95% CI. A negative effect size indicated reduction in cadence; a positive effect size indicated enhancement in cadence. (T: Treadmill)

Figure 10 Forest plot illustrating individual studies evaluating the effects of rhythmic auditory cueing, on time up and go test amongst post-stroke patients. Weighted effect sizes; Hedge’s g (boxes) and 95% C.I (whiskers) are presented. The (Diamond) represents pooled effect sizes and 95% CI. A negative effect size indicated reduction in time up and go test (enhanced postural stability); a positive effect size indicated enhancement in time up and go test (reduced stability).

Table 3 PRISMA Checklist *(*From*:*  Moher D, Liberati A, Tetzlaff J, Altman DG, The PRISMA Group (2009). Preferred Reporting Items for Systematic Reviews and Meta-Analyses: The PRISMA Statement. PLoS Med 6(6): e1000097. doi:10.1371/journal.pmed1000097 *)*

| **Section/topic** | **#** | **Checklist item** | **Reported on page #** |
| --- | --- | --- | --- |
| **TITLE** | | |  |
| Title | 1 | Identify the report as a systematic review, meta-analysis, or both. | 1 |
| **ABSTRACT** | | |  |
| Structured summary | 2 | Provide a structured summary including, as applicable: background; objectives; data sources; study eligibility criteria, participants, and interventions; study appraisal and synthesis methods; results; limitations; conclusions and implications of key findings; systematic review registration number. | 2 |
| **INTRODUCTION** | | |  |
| Rationale | 3 | Describe the rationale for the review in the context of what is already known. | 3-6 |
| Objectives | 4 | Provide an explicit statement of questions being addressed with reference to participants, interventions, comparisons, outcomes, and study design (PICOS). | 6 |
| **METHODS** | | |  |
| Protocol and registration | 5 | Indicate if a review protocol exists, if and where it can be accessed (e.g., Web address), and, if available, provide registration information including registration number. | - |
| Eligibility criteria | 6 | Specify study characteristics (e.g., PICOS, length of follow-up) and report characteristics (e.g., years considered, language, publication status) used as criteria for eligibility, giving rationale. | 6-7 |
| Information sources | 7 | Describe all information sources (e.g., databases with dates of coverage, contact with study authors to identify additional studies) in the search and date last searched. | 8-9 |
| Search | 8 | Present full electronic search strategy for at least one database, including any limits used, such that it could be repeated. | Table 1, 6 |
| Study selection | 9 | State the process for selecting studies (i.e., screening, eligibility, included in systematic review, and, if applicable, included in the meta-analysis). | 8-10 |
| Data collection process | 10 | Describe method of data extraction from reports (e.g., piloted forms, independently, in duplicate) and any processes for obtaining and confirming data from investigators. | Table 1, 6 |
| Data items | 11 | List and define all variables for which data were sought (e.g., PICOS, funding sources) and any assumptions and simplifications made. | 6-7 |
| Risk of bias in individual studies | 12 | Describe methods used for assessing risk of bias of individual studies (including specification of whether this was done at the study or outcome level), and how this information is to be used in any data synthesis. | 7 |
| Summary measures | 13 | State the principal summary measures (e.g., risk ratio, difference in means). | 7-8 |
| Synthesis of results | 14 | Describe the methods of handling data and combining results of studies, if done, including measures of consistency (e.g., I^2^) for each meta-analysis. | 7-8 |

Page 1 of 2

| **Section/topic** | **#** | **Checklist item** | **Reported on page #** |
| --- | --- | --- | --- |
| Risk of bias across studies | 15 | Specify any assessment of risk of bias that may affect the cumulative evidence (e.g., publication bias, selective reporting within studies). | 7-8 |
| Additional analyses | 16 | Describe methods of additional analyses (e.g., sensitivity or subgroup analyses, meta-regression), if done, indicating which were pre-specified. | 7-8 |
| **RESULTS** | | |  |
| Study selection | 17 | Give numbers of studies screened, assessed for eligibility, and included in the review, with reasons for exclusions at each stage, ideally with a flow diagram. | Figure 1, 2, 8 |
| Study characteristics | 18 | For each study, present characteristics for which data were extracted (e.g., study size, PICOS, follow-up period) and provide the citations. | Supplementary Table 2, 8-10 |
| Risk of bias within studies | 19 | Present data on risk of bias of each study and, if available, any outcome level assessment (see item 12). | Figure 2-3, 9-10 |
| Results of individual studies | 20 | For all outcomes considered (benefits or harms), present, for each study: (a) simple summary data for each intervention group (b) effect estimates and confidence intervals, ideally with a forest plot. | 11-16 |
| Synthesis of results | 21 | Present results of each meta-analysis done, including confidence intervals and measures of consistency. | 11-16 |
| Risk of bias across studies | 22 | Present results of any assessment of risk of bias across studies (see Item 15). | Supplementary Table 1, 9 |
| Additional analysis | 23 | Give results of additional analyses, if done (e.g., sensitivity or subgroup analyses, meta-regression [see Item 16]). | 11-16 |
| **DISCUSSION** | | |  |
| Summary of evidence | 24 | Summarize the main findings including the strength of evidence for each main outcome; consider their relevance to key groups (e.g., healthcare providers, users, and policy makers). | 17-23 |
| Limitations | 25 | Discuss limitations at study and outcome level (e.g., risk of bias), and at review-level (e.g., incomplete retrieval of identified research, reporting bias). | 22 |
| Conclusions | 26 | Provide a general interpretation of the results in the context of other evidence, and implications for future research. | 23 |
| **FUNDING** | | |  |
| Funding | 27 | Describe sources of funding for the systematic review and other support (e.g., supply of data); role of funders for the systematic review. | - |

For more information, visit: **www.prisma-statement.org**.

**References**

1 Kobinata, N., Ueno, M., Imanishi, Y. & Yoshikawa, H. Immediate effects of rhythmic auditory stimulation on gait in stroke patients in relation to the lesion site. *Journal of Physical Therapy Science* **28**, 2441-2444 (2016).

2 Ko, B.-W., Lee, H.-Y. & Song, W.-K. Rhythmic auditory stimulation using a portable smart device: short-term effects on gait in chronic hemiplegic stroke patients. *Journal of Physical Therapy Science* **28**, 1538-1543 (2016).

3 Fouad, M. A. & Mousa, G. Effect of rhythmic auditory stimulation on gait in patients with stroke. *Parkinsonism & Related Disorders* **22**, e125 (2016).

4 Song, G.-b. & Ryu, H. J. Effects of gait training with rhythmic auditory stimulation on gait ability in stroke patients. *Journal of Physical Therapy Science* **28**, 1403-1406 (2016).

5 Park, J. H. & Chung, Y. The effects of providing visual feedback and auditory stimulation using a robotic device on balance and gait abilities in persons with stroke: a pilot study. *Physical Therapy Rehabilitation Science* **5**, 125-131, doi:10.14474/ptrs.2016.5.3.125 (2016).

6 Yang, C.-H., Kim, J.-H. & Lee, B.-H. Effects of Real-time Auditory Stimulation Feedback on Balance and Gait after Stroke: a Randomized Controlled Trial. *Journal of Experimental Stroke & Translational Medicine* **9**, 1-5 (2016).

7 Yoon, S. K. & Kang, S. H. Effects of inclined treadmill walking training with rhythmic auditory stimulation on balance and gait in stroke patients. *Journal of Physical Therapy Science* **28**, 3367-3370 (2016).

8 Brasileiro, A. *et al.* Influence of visual and auditory biofeedback on partial body weight support treadmill training of individuals with chronic hemiparesis: a randomized controlled clinical trial. *European journal of physical and rehabilitation medicine* **51**, 49-58 (2015).

9 Shin, Y.-K., Chong, H. J., Kim, S. J. & Cho, S.-R. Effect of rhythmic auditory stimulation on hemiplegic gait patterns. *Yonsei medical journal* **56**, 1703-1713 (2015).

10 Ki, K.-I., Kim, M.-S., Moon, Y. & Choi, J.-D. Effects of auditory feedback during gait training on hemiplegic patients’ weight bearing and dynamic balance ability. *Journal of physical therapy science* **27**, 1267-1269 (2015).

11 Jung, K. *et al.* Effects of gait training with a cane and an augmented pressure sensor for enhancement of weight bearing over the affected lower limb in patients with stroke: a randomized controlled pilot study. *Clin. Rehabil.* **29**, 135-142 (2015).

12 Yoon, S. & Kang, S. Effects of Inclined Treadmill Walking Training with Rhythmic Auditory Stimulation on Balance and Gait in Stroke Patients: A pilot study. *Journal of The Korean Society of Integrative Medicine* **3**, 69-78 (2015).

13 Park, J., Park, S.-y., Kim, Y.-w. & Woo, Y. Comparison between treadmill training with rhythmic auditory stimulation and ground walking with rhythmic auditory stimulation on gait ability in chronic stroke patients: A pilot study. *NeuroRehabilitation* **37**, 193-202 (2015).

14 Oh, Y.-s., Kim, H.-s. & Woo, Y.-k. Effects of rhythmic auditory stimulation using music on gait with stroke patients. *Physical Therapy Korea* **22**, 81-90 (2015).

15 Hashiguchi, Y. *et al.* Effect of rhythmic auditory stimulation on gait parameters and gait emg in patients with hemiplegia after stroke. *Gait. Posture.* **39**, S139 (2014).

16 Cha, Y., Kim, Y. & Chung, Y. Immediate effects of rhythmic auditory stimulation with tempo changes on gait in stroke patients. *Journal of physical therapy science* **26**, 479-482 (2014).

17 Suh, J. H. *et al.* Effect of rhythmic auditory stimulation on gait and balance in hemiplegic stroke patients. *NeuroRehabilitation* **34**, 193-199 (2014).

18 Cha, Y., Kim, Y., Hwang, S. & Chung, Y. Intensive gait training with rhythmic auditory stimulation in individuals with chronic hemiparetic stroke: A pilot randomized controlled study. *NeuroRehabilitation* **35**, 681-688 (2014).

19 Wright, R. L. *et al.* Metronome-cued stepping in place after hemiparetic stroke: Comparison of a one-and two-tone beat. *ISRN Rehabilitation* **2013** (2013).

20 Lee, S. H., Lee, K. J. & Song, C. H. Effects of rhythmic auditory stimulation (RAS) on gait ability and symmetry after stroke. *Journal of Physical Therapy Science* **24**, 311-314 (2012).

21 Chouhan, S. & Kumar, S. Comparing the effects of rhythmic auditory cueing and visual cueing in acute hemiparetic stroke. *International Journal of Therapy & Rehabilitation* **19** (2012).

22 Muto, T., Herzberger, B., Hermsdoerfer, J., Miyake, Y. & Poeppel, E. Interactive cueing with walk-mate for hemiparetic stroke rehabilitation. *J. Neuroeng. Rehabil.* **9**, 58 (2012).

23 Jung-Hee, K. *et al.* Effects of the combination of rhythmic auditory stimulation and task-oriented training on functional recovery of subacute stroke patients. *Journal of physical therapy science* **24**, 1307-1313 (2012).

24 Kim, J.-s. & Oh, D.-w. Home-based auditory stimulation training for gait rehabilitation of chronic stroke patients. *Journal of Physical Therapy Science* **24**, 775-777 (2012).

25 Jung, J., Cho, K., Shim, S., Yu, J. & Kang, H. The effects of integrated visual and auditory stimulus speed on gait of individuals with stroke. *Journal of Physical Therapy Science* **24**, 881-883 (2012).

26 Johannsen, L. *et al.* Seated bilateral leg exercise effects on hemiparetic lower extremity function in chronic stroke. *Neurorehabil. Neural. Repair.* **24**, 243-253 (2010).

27 Park, I. M., Oh, D. W., Kim, S. Y. & Choi, J. D. Clinical feasibility of integrating fast-tempo auditory stimulation with self-adopted walking training for improving walking function in post-stroke patients: a randomized, controlled pilot trial. *Journal of Physical Therapy Science* **22**, 295-300 (2010).

28 Pelton, T. A., Johannsen, L., Chen, H. & Wing, A. M. Hemiparetic stepping to the beat: asymmetric response to metronome phase shift during treadmill gait. *Neurorehabil. Neural. Repair.* **24**, 428-434 (2010).

29 Roerdink, M. *et al.* Rhythm perturbations in acoustically paced treadmill walking after stroke. *Neurorehabil. Neural. Repair.* **23**, 668-678 (2009).

30 Hayden, R., Clair, A. A., Johnson, G. & Otto, D. The effect of rhythmic auditory stimulation (RAS) on physical therapy outcomes for patients in gait training following stroke: a feasibility study. *International Journal of Neuroscience* **119**, 2183-2195 (2009).

31 Roerdink, M., Lamoth, C. J., Kwakkel, G., Van Wieringen, P. C. & Beek, P. J. Gait coordination after stroke: benefits of acoustically paced treadmill walking. *Physical Therapy* **87**, 1009 (2007).

32 Argstatter, H., Hillecke, T., Thaut, M. & Bolay, H. Musiktherapie in der neurologischen Rehabilitation. *Neurol Rehabil* **13**, 42-48 (2007).

33 Thaut, M. H. *et al.* Rhythmic auditory stimulation improves gait more than NDT/Bobath training in near-ambulatory patients early poststroke: a single-blind, randomized trial. *Neurorehabil. Neural. Repair.* **21**, 455-459, doi:10.1177/1545968307300523 (2007).

34 Schauer, M. & Mauritz, K. H. Musical motor feedback (MMF) in walking hemiparetic stroke patients: randomized trials of gait improvement. *Clin Rehabil* **17**, 713-722, doi:10.1191/0269215503cr668oa (2003).

35 Thaut, M. H., McIntosh, G. & Rice, R. Rhythmic facilitation of gait training in hemiparetic stroke rehabilitation. *Journal of the neurological sciences* **151**, 207-212 (1997).

36 Prassas, S., Thaut, M., McIntosh, G. & Rice, R. Effect of auditory rhythmic cuing on gait kinematic parameters of stroke patients. *Gait. Posture.* **6**, 218-223 (1997).

37 Thaut, M. H., McIntosh, G. C., Prassas, S. G. & Rice, R. R. Effect of rhythmic auditory cuing on temporal stride parameters and EMG. Patterns in hemiparetic gait of stroke patients. *Journal of Neurologic Rehabilitation* **7**, 9-16 (1993).

38 Sangita, K. & Remya, N. The Effect of Rhythmic Auditory Stimulation in Gait Training among Stroke Patients. *Indian Journal of Physiotherapy and Occupational Therapy-An International Journal* **10**, 61-66 (2016).
